# Anthranilic acid, the new player in the ensemble of aromatic residue labeling precursor compounds

**DOI:** 10.1007/s10858-017-0129-2

**Published:** 2017-08-31

**Authors:** Julia Schörghuber, Leonhard Geist, Marilena Bisaccia, Frederik Weber, Robert Konrat, Roman J. Lichtenecker

**Affiliations:** 1 Institute of Organic Chemistry, University of Vienna, Währingerstr. 38, 1090 Vienna, Austria; 2 Christian Doppler Laboratory for High-Content Structural Biology and Biotechnology, Department of Structural and Computational Biology, Max F. Perutz Laboratories, University of Vienna, Dr-Bohr-Gasse 9, 1030 Vienna, Austria

**Keywords:** Isotope labeling, Protein NMR, Tryptophan, Anthranilic acid

## Abstract

The application of metabolic precursors for selective stable isotope labeling of aromatic residues in cell-based protein overexpression has already resulted in numerous NMR probes to study the structural and dynamic characteristics of proteins. With anthranilic acid, we present the structurally simplest precursor for exclusive tryptophan side chain labeling. A synthetic route to ^13^C, ^2^H isotopologues allows the installation of isolated ^13^C–^1^H spin systems in the indole ring of tryptophan, representing a versatile tool to investigate side chain motion using relaxation-based experiments without the loss of magnetization due to strong ^1^J_CC_ and weaker ^2^J_CH_ scalar couplings, as well as dipolar interactions with remote hydrogens. In this article, we want to introduce this novel precursor in the context of hitherto existing techniques of in vivo aromatic residue labeling.

**Electronic supplementary material:**

The online version of this article (doi:10.1007/s10858-017-0129-2) contains supplementary material, which is available to authorized users.

## Introduction

The progress in stable isotope labeling has driven the field of protein NMR spectroscopy, ever since the first spectra of small randomly deuterated or uniformly ^15^N-labeled proteins have been recorded (Campbell 2013). Numerous experimental techniques have been developed in the meantime to address a wide range of structure-biological questions (Lian and Middleton 2001; Ohki and Kainosho 2008; Zhang and van Ingen 2016). However, poor signal resolution and complex magnetization transfer causes severe problems in the case of high molecular weight target proteins, which can only be addressed using highly defined patterns of heavy isotopes (^13^C, ^15^N, ^2^H). Early examples of selective stable isotope labeling almost exclusively focused on aliphatic side chains (Goto and Kay 2000; Kerfah et al. 2015). Their high abundance in the core of globular proteins, as well as their intense NMR signals due to the threefold symmetry of a methyl group rotation result in straightforward data acquisition for structure calculation. These aliphatic side chains play an important role in protein folding, creating a well-defined hydrophobic microenvironment for substrate binding and solvent exclusion.

Although some very early examples can be found in literature (Wüthrich and Wagner 1975), it was only in recent years, that studies of aromatic side chains increasingly attracted the attention of the biomolecular NMR community. Aromatic side chains provide valuable additional restraints for structure calculation and exhibit a number of characteristics, which turn them into important sensors to study enzymatic mechanisms or binding events. They are prone to participate in non-covalent π–π or π–cation interactions, which significantly contribute to the protein’s tertiary structure (Dougherty 1996). Aromatic residues additionally control enzymatic reaction trajectories by stabilizing charged intermediates or direct structural changes to close hydrophobic cavities and protect them from water molecules. Due to their distinct physicochemical properties, aromatic side chains play a very prominent role at binding interfaces to other proteins, nucleic acids, lipid membranes or small ligands (Moreira et al. 2013; Rahman et al. 2015). These binding events, as well as conformational changes during enzymatic catalysis, are frequently connected to extensive aromatic side chain motion in a wide range of time scales from picoseconds to seconds.

Side chain dynamics, induced by binding events or changes in temperature or pressure, have been studied using different NMR based techniques (Boehr et al. 2006). The corresponding experiments, such as longitudinal- and transverse-^13^C-relaxation (Weininger et al. 2012a, 2014a; Kasinath et al. 2015), the ^13^C-TROSY-Carr-Purcell-Meiboom-Gill (CPMG) experiment (Weininger et al. 2012b), the ^13^C-TROSY-rotating frame relaxation (R_1ρ_) sequence (Weininger et al. 2014b), intra-residue NOE interpretation (Miyanoiri et al. 2011), or the recording of aromatic residual dipolar couplings (Sathyamoorthy et al. 2013) have been optimized for applications in aromatic spin systems. These methods resulted in the calculation of rate constants, energy barriers, as well as activation volumes. As mentioned above, the data of such experiments can only be interpreted when spectra have been simplified and magnetization transfer pathways optimized using defined isotope distributions in the protein samples.

Two main strategies can be pursued to generate defined isotope patterns in a protein of interest: In cell-free overexpression methods all components needed for the transcription and the translation process are provided to an in vitro system. This approach is well suited to generate proteins, which contain posttranslational modifications or modified amino acids, as well as proteins that are toxic to in vivo systems. Selective labeling devoid of cross labeling to other residues is assured, but the method requires the application of labeled amino acids (Torizawa et al. 2005; Kainosho et al. 2006; Takeda et al. 2010; Miyanoiri et al. 2011). Moreover, cell-free protein expression exhibits major shortcomings in providing high protein yields and is considerably expensive (Staunton et al. 2006).

**Scheme 1:**
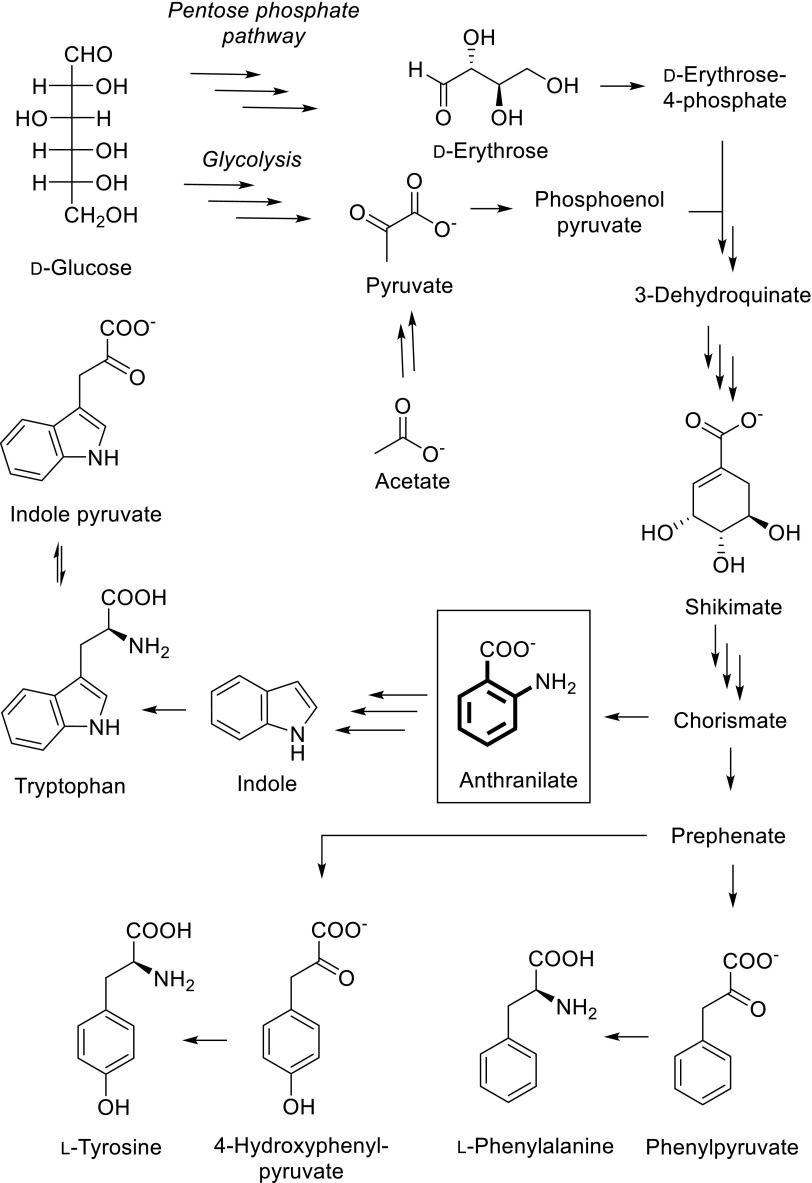
Overview of aromatic residue labeling precursor compounds in *E. coli* overexpression media

The second strategy features isotope labeled precursor compounds as additives in the growth medium of an overexpressing microorganism, which has been established as the method of choice to yield in high concentrations of the corresponding labeled molecular targets (Hoogstraten and Johnson 2008). Potential pitfalls include poor precursor uptake or cross labeling to other residues. Scheme 1 summarizes the main metabolites, which have been used to overexpress proteins containing labeled phenylalanine, tyrosine or tryptophan residues. Certain ^13^C-patterns of D-glucose (Teilum et al. 2006; Lundström et al. 2007), acetate (Wand et al. 1995), erythrose (Kasinath et al. 2013; Weininger 2017), pyruvate (Guo et al. 2009; Milbradt et al. 2015), or glycerol (LeMaster and Kushlan 1996; Ahlner et al. 2015) have been applied as carbon sources to avoid direct ^13^C–^13^C coupling in the target residues. These compounds are commercially available and additional organic synthesis is usually not required. However, the costs are considerable regarding the concentrations needed for high incorporation levels (up to 3 g/L medium). These methods partially exhibit poor selectivity, as the corresponding isotope sources represent early metabolic intermediates residing upstream of the shikimate pathway. This inevitably leads to the undesired loss of heavy isotopes at metabolic branches. The metabolites of the shikimate pathway 3-dehydroquinate to prephenate feature multiple chiral centers and are therefore rather too complex to be considered as isotope containing targets in economic multistep organic synthesis. In only one example unlabeled shikimate was thus used to introduce protonated aromatic residues into an otherwise deuterated protein (Rajesh et al. 2003). As soon as the aromatic ring system is biosynthetically formed, the corresponding metabolic intermediates represent much more attractive target molecules for organic synthesis. In rare cases, aromatic amino acids have been utilized in combination with in vivo overexpression media for selective labeling/unlabeling (Vuister et al. 1994; Kelly et al. 1999; Wang et al. 1999). However, these examples often suffer from poor metabolic uptake and increased effects on metabolic network regulation, since many of the enzymes involved are inhibited by the final product of the corresponding biosynthetic pathway. These methods additionally lack the possibility of simple uniform ^15^N labeling by overexpressing the target protein in presence of ^15^N-ammonium chloride, which is beneficial for many NMR applications. α-Ketoacids can be considered as late metabolic intermediates and their use as precursors for aliphatic residue labeling has a long history due to the effective uptake and the straightforward aminotransferase-catalyzed conversion to the target amino acids (Goto et al. 1999; Lichtenecker et al. 2004, 2013a). In a similar approach for aromatic residue labeling, phenylpyruvate has been shown to be an effective precursor for phenylalanine, whereas the use of 4-hydroxyphenylpyruvate leads to tyrosine labeling (Lichtenecker et al. 2013b). Isotopologues of indole have been used to selectively label tryptophan side chains (Schörghuber et al. 2015; Rodriguez-Mias and Pellecchia 2003). Alternatively, indole pyruvate can be applied for backbone Trp-labeling, which is the first intermediate in the tryptophan degradation pathway, but is effectively converted to the target amino acid due to the highly reversible character of the Trp-aminotransferase.

**Scheme 2:**
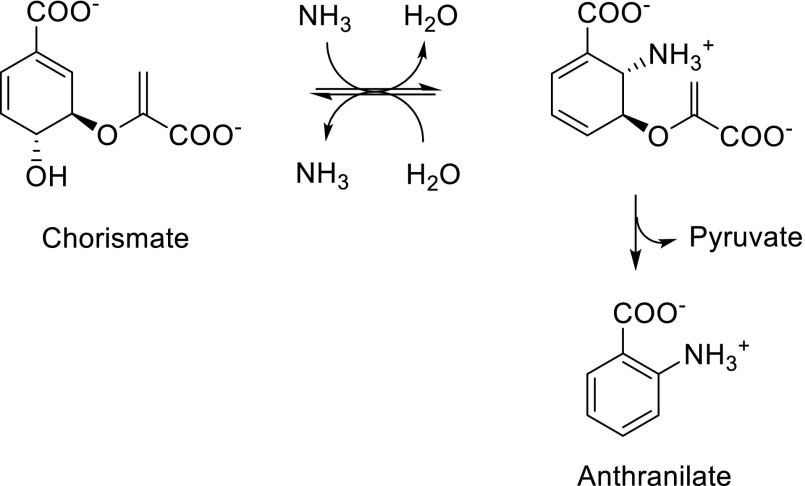
Anthranilate synthase catalyzed conversion of chorismate to anthranilate

The structurally simplest precursor, which holds promise to result in exclusive tryptophan labeling is anthranilic acid. Although ^15^N-isotopologues of this compound have been used in the past to produce [1-^15^N]L-tryptophan by fermentation (Liu et al. 2009) and protocols for the synthesis of different stable isotope patterns have been reported (van Liemt et al. 1996), methods to apply anthranilic acid as a direct metabolic precursor in protein overexpression media are still not available, at least to our knowledge. We hypothesized that none of the heavy isotopes present when using anthranilic acid as a tryptophan precursor should be metabolized back into the shikimic acid pathway. Since the second reaction step in the anthranilate synthase (*EC 4.1.3.27*) catalyzed conversion of chorismate to anthranilate is an irreversible process (elimination of pyruvate, Scheme 2), we expected exclusive labeling of the tryptophan target residues without any cross labeling to phenylalanines and tyrosines.

## Materials and methods

### Organic synthesis

Detailed protocols for the synthesis of the anthranilic acid isotopologues [^15^N]anthranilic acid **4**, 3,5-dideuterio[4,6-^13^C_2_]anthranilic acid **11**, and 4,6-dideuterio[5-^13^C]anthranilic acid **18**, as well as NMR and MS characterization of intermediates and final products are provided in the supporting information.

### Protein overexpression

#### His-tagged GB1

The pET-M11 plasmid containing the sequence for a his-tagged GB1 protein (H6-GB1) was transformed into an *E. coli* BL21(DE3) strain. Bacterial cultures were grown in LB medium and then transferred to a M9 minimal medium supplemented either with unlabeled NH_4_Cl (1 g/L) and increasing amounts of [^15^N]anthranilic acid **4** to acquire selectively labeled protein or with ^15^NH_4_Cl (1 g/L) to acquire uniformly labeled protein (Marley et al. 2001). Protein overexpression was induced by the addition of isopropyl-β-D-thiogalactopyranosid and pursued overnight. The crude proteins were purified using Ni^2+^ affinity chromatography. NMR samples contained 1 mM protein and 10% D_2_O.

#### Brd4-BD1

Recombinant human Brd4-BD1 (bromodomain 1 of Bromodomain containing protein 4) was expressed in *E. coli* BL21(DE3) containing an N-terminal TEV-cleavable His6-tag (the expression plasmid was kindly provided by Boehringer Ingelheim). Uniformly ^15^N/selectively ^13^C,^2^H-tryptophan labeled H6-TEV-Brd4-BD1 was expressed following the expression protocol for efficient isotopic labeling of recombinant proteins using a fourfold cell concentration in M9 minimal medium supplemented with ^15^NH_4_Cl (1 g/L) and 3,5-dideuterio[4,6-^13^C_2_]anthranilic acid **11** (50 mg/L) (Marley et al. 2001). Uniformly ^15^N / uniformly ^13^C-labeled Brd4-BD1 was overexpressed using 1 g/L ^15^NH_4_Cl and 3 g/L ^13^C_6_-glucose in the minimal medium. Cells were harvested by centrifugation, lysed by sonication and the lysates were subsequently centrifuged. Proteins were purified from the resulting supernatant by Ni^2+^ affinity chromatography. The purified protein was treated with TEV protease and again loaded onto a Ni^2+^ column to bind the cleaved His6-tag and the His6-tagged TEV protease. The flow-through containing Brd4-BD1 was concentrated and purified on a gel filtration column. NMR samples of Brd4-BD1 were prepared in sodium phosphate buffer containing 0.4–3 mM protein and 10% D_2_O.

Comprehensive information concerning the overexpression of labeled H6-GB1 and Brd4-BD1 is given in the supporting information.

### Protein NMR spectroscopy

NMR spectra of H6-GB1 and Brd4-BD1 were acquired at 298 K on a Bruker Avance 3 HD + 600 MHz spectrometer. The system was equipped with a RT 5 mm TXI probe. Spectra were processed using NMR Pipe (Delaglio et al. 1995) and analyzed using the Sparky software (Goddard and Kneller). Methodological details concerning the quantification of H6-GB1 labeling using compound **4**, as well as an evaluation of Trp-^13^C-labeling of Brd4-BD1 using compound **11** are reported in the supporting information.

## Results and discussion

**Scheme 3:**
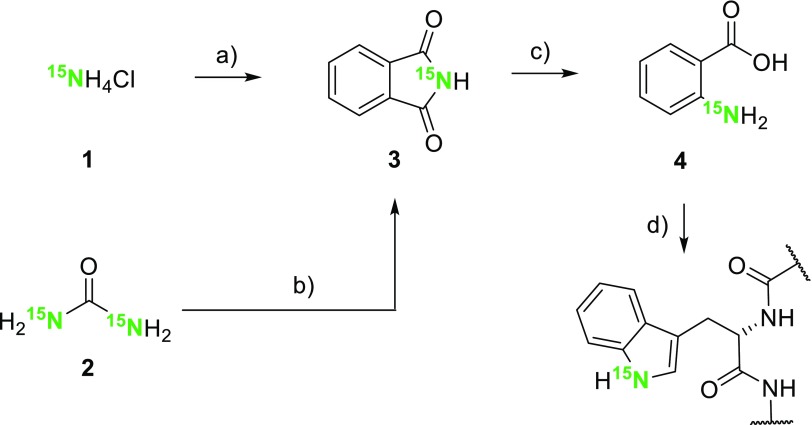
Synthesis of [^15^N]anthranilic acid **4** and its application to selectively label Trp-residues. **a** Phthalic anhydride, 250 °C, DMAP, 66%; **b** phthalic anhydride, xylenes, 140 °C, 84%; **c** NaOH, Br_2_, 53%; **d** *E. coli* protein overexpression, D-glucose, NH_4_Cl

**Fig. 1:**
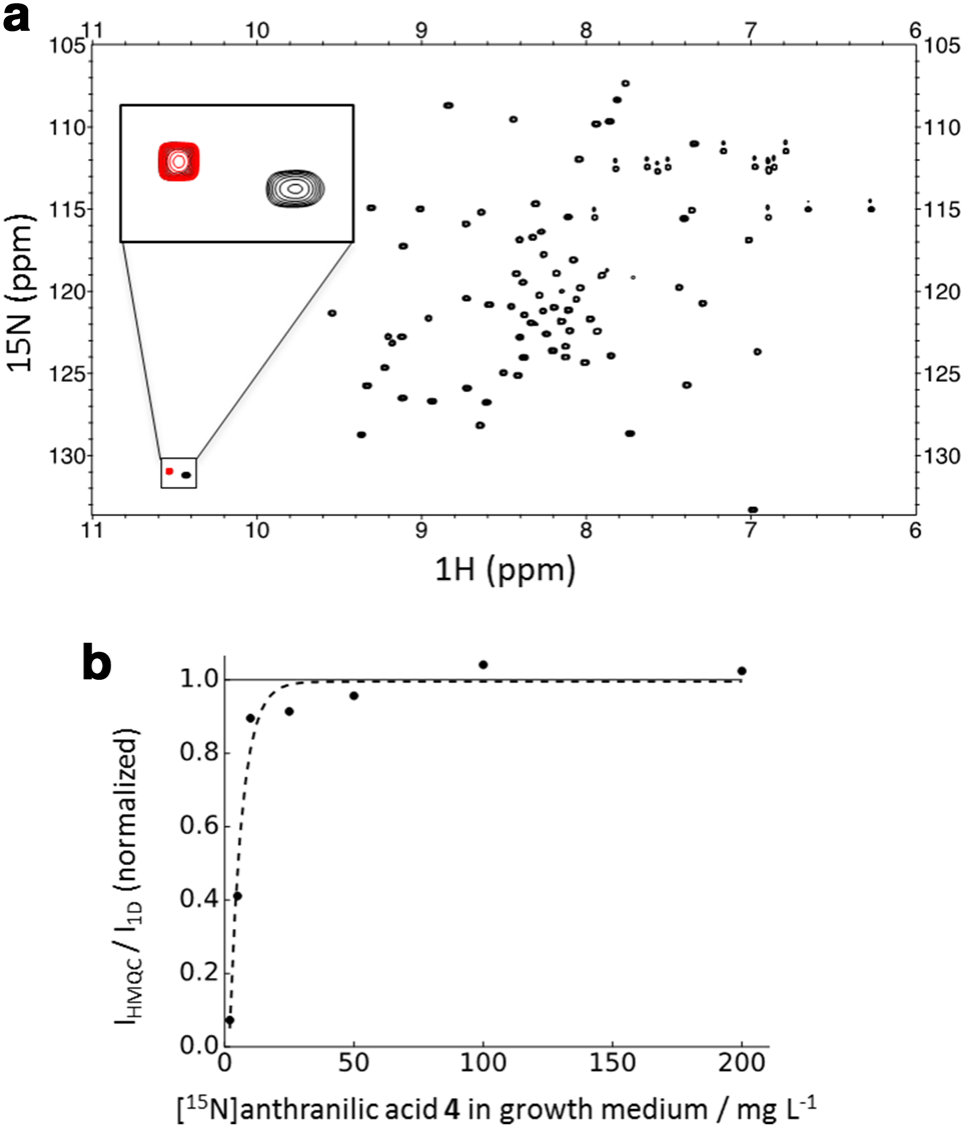
**a** ^15^N-HSQC spectra overlay of the uniformly ^15^N-labeled (*black*) and tryptophan side chain ^15^N labeled (*red*) H6-GB1 protein; **b** normalized signal intensity versus precursor concentration plot showing data for the overexpression of H6-GB1 in presence of 2, 5, 10, 25, 50, 100 and 200 mg/L of precursor **4**. The intensities have been normalized to a uniformly ^15^N-labeled sample (1 on* y*-axis)

In order to investigate the uptake of anthranilic acid by the expressing *E. coli* strain, we prepared [^15^N]anthranilic acid **4** in a two-step sequence starting from ^15^NH_4_Cl **1** or [^15^N]urea **2** (Scheme 3) (Hijji and Benjamin 2003). An *E. coli* strain, grown in the presence of compound **4**, overexpressed the exclusively tryptophan-^15^N histidine-tagged G-binding protein domain (H6-GB1). The resulting ^15^N-HSQC spectrum is devoid of any other signals, except the one caused by the only ^1^H_ε_–^15^N_ε_ Trp side chain spin system in the target protein (Fig. 1). [^15^N]Anthranilic acid **4** was applied to correlate the precursor concentrations in the growth medium to the protein isotope incorporation rates. The corresponding plot in Fig. 1b displays normalized ^15^N-HSQC signal intensities of H6-GB1 overexpressed in presence of different precursor **4** concentrations. Signal intensities were compared to the intensity found for the ^1^H_ε_–^15^N_ε_ Trp side chain signal in uniformly ^15^N-labeled H6-GB1 (experimental details are given in the supporting information). A signal intensity of >90% at a concentration of 10 mg/L indicates the effective metabolic uptake and conversion of anthranilic acid by the overexpressing host organism.

**Scheme 4:**
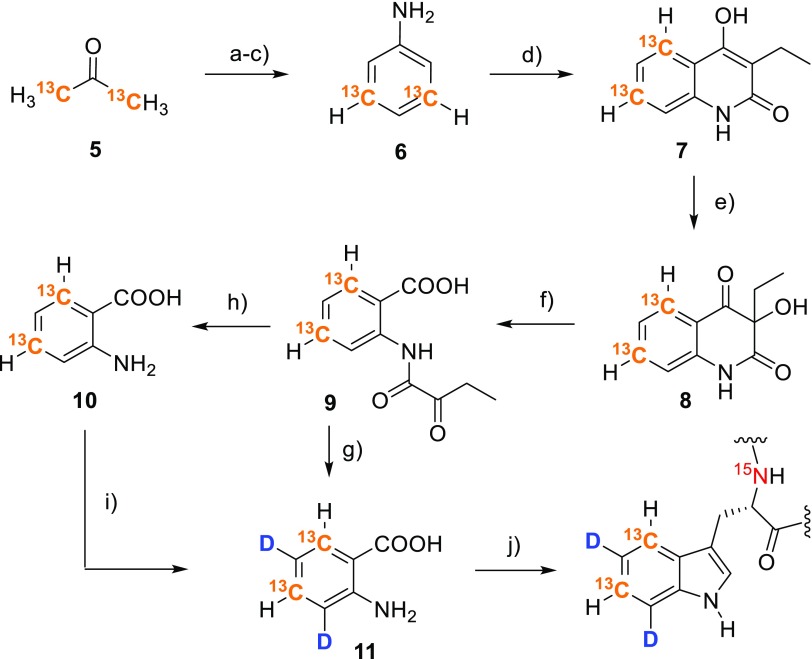
Synthesis of 3,5-dideuterio[4,6-^13^C_2_]anthranilic acid **11** and its application to selectively label Trp-residues. **a** Sodium nitromalonaldehyde monohydrate, aqu. NaOH, then 6N HCl, 63%; **b** potassium *tert*-butoxide, dimethylformamide, 5-chloro-1-phenyl-*1H*-tetrazole, 74%; **c** Pd/C, methanol, toluene, H_2_, H-cube^®^, 97%; **d** diethyl ethylmalonate, 250–270 °C, then toluene and aqu. NaOH, 77%; **e** peroxyacetic acid in acetic acid, aqu. NaOH; **f** paraperiodic acid, ethanol, 75% over steps (**ef**); **g** DCl/D_2_O, 94%; **h** HCl/H_2_O, 91%; **i** microwave irradiation, DCl/D_2_O, 92%; **j** *E. coli* protein overexpression, D-glucose, ^15^NH_4_Cl

These results encouraged us to develop a synthetic route to anthranilic acid isotopologues possessing ^13^C in the aromatic ring (Scheme 4). Starting from [1,3-^13^C_2_]acetone **5**, we assembled the aromatic system through reaction with nitromalonaldehyde (Viswanatha and Hruby 1979). A two-step deoxygenation/reduction sequence yielded [3,5-^13^C_2_]aniline **6** (Musliner and Gates 1966), which was transformed into anthranilic acid following a literature protocol (Kafka et al. 2013). Hydrolysis of N-(α-ketoacyl)anthranilic acid **9** in DCl/D_2_O yielded 3,5-dideuterio[4,6-^13^C_2_]anthranilic acid **11**. The ^2^H-pattern can be installed using mild reaction conditions, since the electron donating amino substituent directs the reaction outcome to deuteration in the desired *ortho* and *para* positions.

**Scheme 5:**
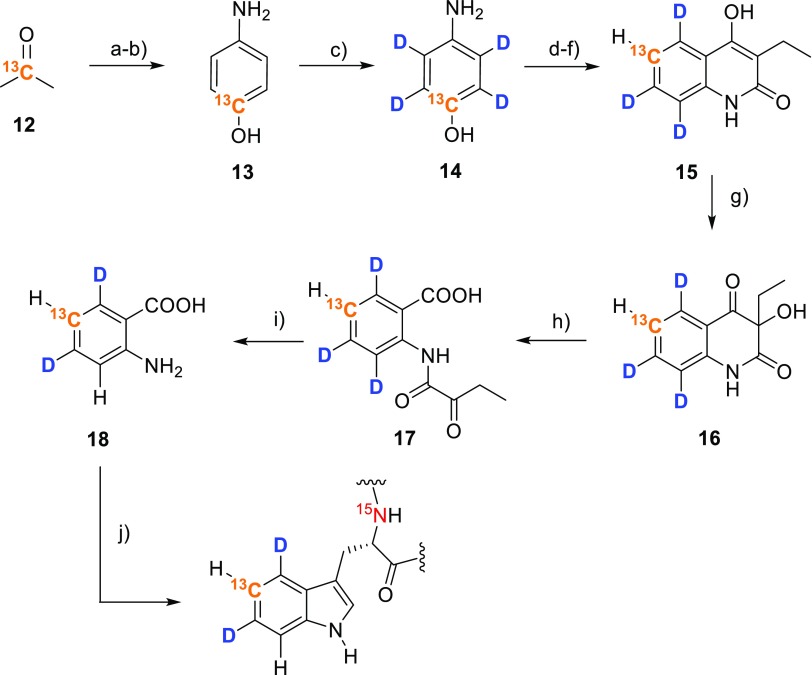
Synthesis of 4,6-dideuterio[5-^13^C]anthranilic acid **18** and its application to selectively label Trp-residues. **a** Sodium nitromalonaldehyde monohydrate, aqu. NaOH, then 6N HCl, 60%; **b** Pd/C, H_2_, H-cube^®^, methanol, toluene, 95%; **c** HCl/D_2_O, MW irradiation, 180 °C, 7 h, 90%; **d** potassium *tert*-butoxide, dimethylformamide, 5-chloro-1-phenyl-*1H*-tetrazole, 75%; **e** Pd/C 10%, MeOH, toluene, H_2_, H-cube^®^, 95%; **f** diethyl ethylmalonate, heat, then toluene and aqu. NaOH, 69%; **g** peroxyacetic acid in acetic acid, aqu. NaOH; **h** paraperiodic acid, ethanol, 72% over steps (**g, h**); **i** HCl/H_2_O, 90%; **j** *E. coli* protein overexpression, D-glucose, ^15^NH_4_Cl

**Fig. 2:**
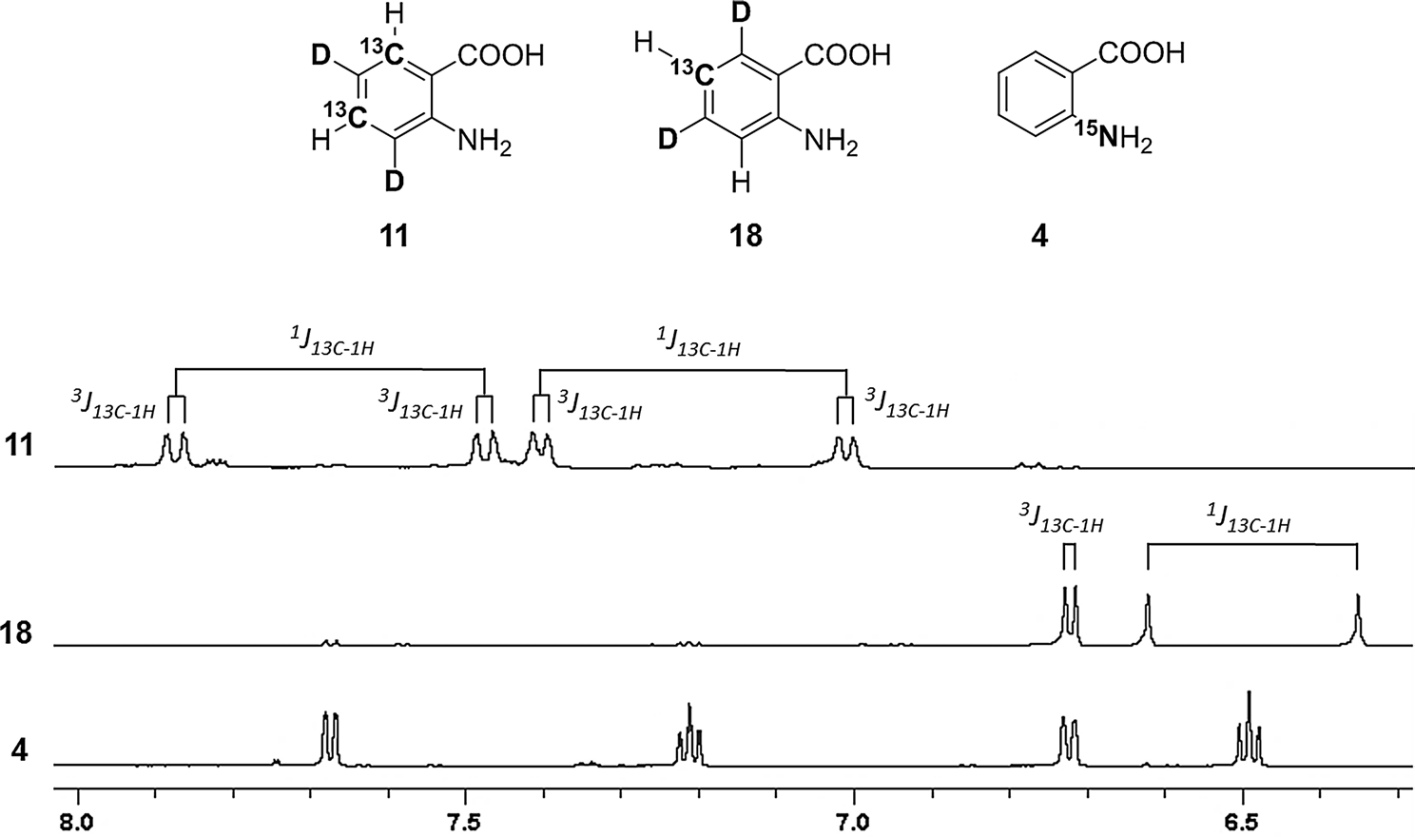
^1^H-NMR spectral region of aromatic protons showing the corresponding ^13^C–^1^H couplings of 3,5-dideuterio [4,6-^13^C_2_]anthranilic acid **11**, 4,6-dideuterio [5-^13^C]anthranilic acid **18** and [^15^N]anthranilic acid **4**, respectively

Using a similar route, but starting from [2-^13^C]acetone **12** allows for the synthesis of the 4,6-dideuterio[5-^13^C] isotopologue of the target compound (**18**, Scheme 5). In this case ^2^H is introduced by perdeuteration of [^13^C]aminophenol in acidic D_2_O at 180 °C at prolonged reaction times (**13**→**14**). The labeling patterns of compounds **11** and **18** are well defined (Fig. 2) and feature isolated ^13^C–^1^H spin systems, which can be effectively applied for the elucidation of tryptophan dynamics using ^13^C transverse relaxation dispersion based methods.

**Fig. 3:**
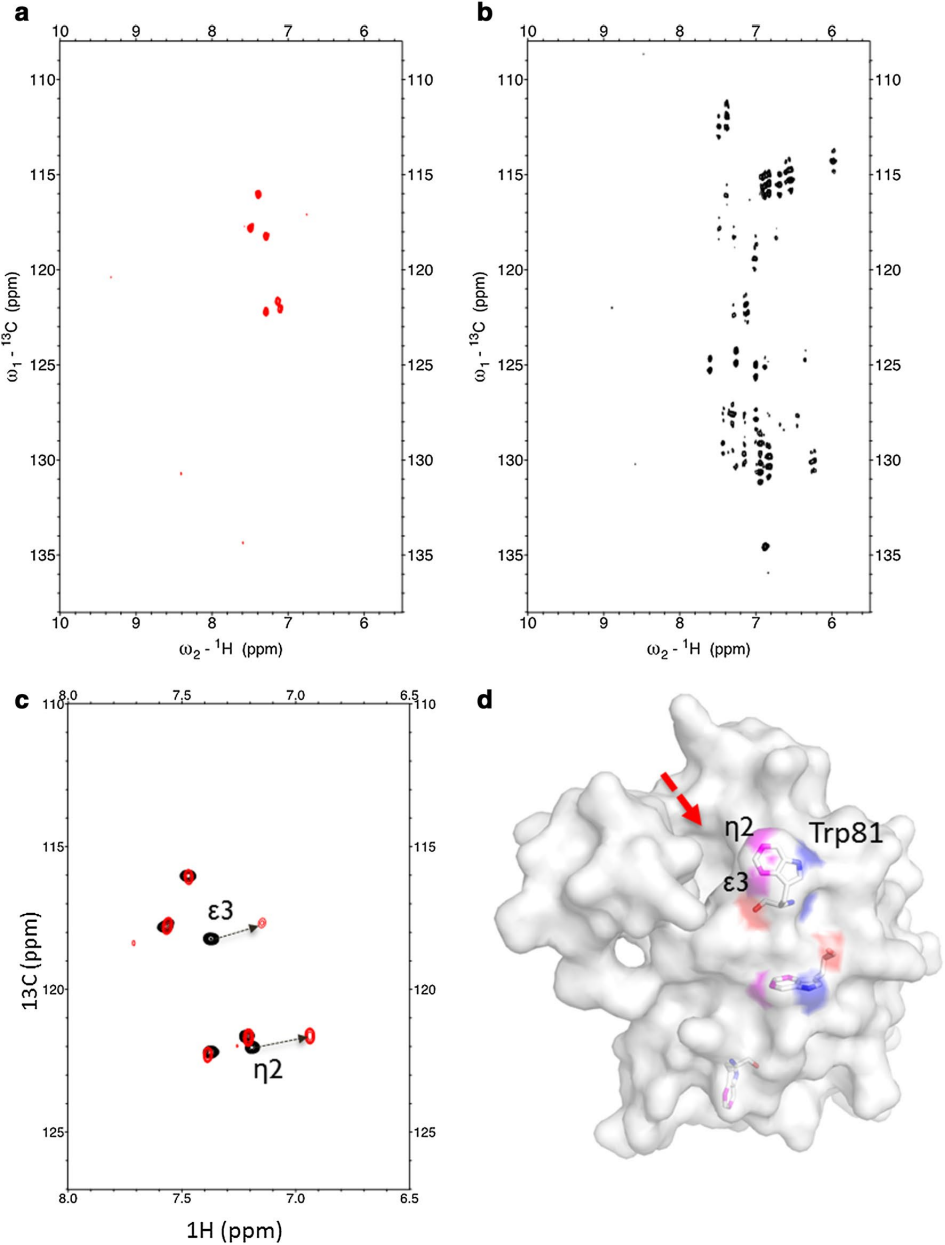
**a** ^13^C-HSQC of Brd4-BD1 overexpressed in the presence of 3,5-dideuterio[4,6-^13^C_2_]anthranilic acid **11; b** ^13^C-HSQC of Brd4-BD1 overexpressed in the presence of [^13^C_6_]D-glucose; **c** ^13^C-HSQC of selectively Trp-^13^C/^2^H labeled Brd4-BD1 (*black*) and upon addition of the Brd4-BD1 binding ligand (*red*); **d** X-ray structure of apo-Brd4-BD1 (4LYI) (Lucas et al. 2013) depicting the three tryptophan residues. Trp81 is part of the Brd4-BD1 binding pocket (*red arrow*)

3,5-Dideuterio[4,6-^13^C_2_]anthranilic acid **11** was supplemented to the *E. coli* growth medium to overexpress the recombinant human bromodomain 1 of Bromodomain containing protein 4 featuring an N-terminal TEV-cleavable His6-tag (H6-TEV-Brd4-BD1) (see “Materials and methods” section for details). BRD4 is linked to diverse human malignancies and has been shown to regulate the expression of *MYC* (Andrews et al. 2017). The *MYC* gene encodes a transcription factor, which plays a decisive part in cancer development and proliferation. The corresponding bromodomain was chosen as a model to demonstrate exclusive Trp labeling by our novel precursor compound, as well as give a first representative of its potential use. The resulting ^13^C-HSQC spectra showed a total number of six signals, caused by the ^13^C–^1^H_ε3_ and ^13^C–^1^H_η2_ couplings of the three tryptophan residues present in the corresponding sequence (Fig. 3a). Comparing this HSQC with spectra obtained from a uniformly ^13^C labeled sample, which was overexpressed in presence of ^13^C_6_-glucose, reveals the value of our labeling approach in terms of well-defined signals of high intensity in absence of signal splitting or line shape distortion due to scalar couplings (Fig. 3b). The ^1^H-^15^N HSQC of the Brd4-BD1 sample which was labeled using ^15^NH_4_Cl and compound **11** showed the complete absence of Trp-Nε_2_ resonances, given that the precursor 3,5-dideuterio[4,6-^13^C_2_]anthranilic acid **11** carries an unlabeled amino-group (see supporting information).

This data confirmed our initial assumption that anthranilic acid serves as a selective tryptophan precursor with quantitative incorporation levels devoid of any cross-labeling to the other aromatic residues. Addition of a ligand (kindly provided by Boehringer Ingelheim) targeting the binding site of Brd4-BD1 for acetylated lysines in histones resulted in an upfield shift of the Trp81 signals (Fig. 3c). This residue is positioned in the bromodomain binding-cleft, thus playing a central role in protein–ligand interactions (Fig. 3d) (Zhang et al. 2015).

## Conclusions

In summary, the *E. coli* overexpression of model proteins in presence of labeled anthranilic acid demonstrated both, high labeling selectivity for the target Trp-residues, as well as highly effective precursor uptake by the overexpressing host organism with quantitative isotope incorporation levels at low precursor concentrations (10–15 mg/L overexpression medium). Our novel labeling technique represents an advanced development of hitherto applied methods using early metabolic intermediates as heavy isotope sources. Recently, isotopologues of erythrose have been introduced to direct ^13^C labeling into defined atomic positions of the aromatic side-chains in phenylalanine, tyrosine and tryptophan (Kasinath et al. 2015; Weininger 2017). In the corresponding protocols, limited isotope incorporation levels in the range of 50–70% for tryptophan, as well as partial isotope scrambling to undesired atomic positions in the aromatic rings have been reported. On the contrary, anthranilic acid is metabolized to the target amino acid in a well-defined way without losing labeling selectivity at the major metabolic intersection points of the shikimate pathway. Our novel precursor completes our toolbox of isotope labeled compounds to transfer ^13^C/^2^H-patterns onto the aromatic side chains of phenylalanine and tyrosine (Lichtenecker et al. 2013b), tryptophan (Schörghuber et al. 2015), as well as histidine (Schörghuber et al. 2017), with every precursor targeting one specific type of residue. This residue selective isotope incorporation cannot be achieved using the early metabolic precursors mentioned above, but is of major importance in the case of high molecular weight protein samples, when signal overlap in the aromatic region becomes a serious issue.

Efficient metabolic in vivo conversion has also been observed for the selective tryptophan precursor indol, which we presented in a recent publication (Schörghuber et al. 2015). However, the synthetic routes to anthranilic acid isotopologues from the present study offer clear advantages in terms of yields and robustness and are thus preferred in cases where the γ and δ_1_ positions are not targeted for ^13^C labeling. The synthetic routes to 3,5-dideuterio[4,6-^13^C_2_]anthranilic acid **11** and 4,6-dideuterio[5-^13^C]anthranilic acid **18** have been optimized to hold down costs for isotope sources and reagents, while at the same time minimizing synthetic efforts. Both precursors are accessible in gram scale and can be stored and handled as bench-stable solids. Considering the listed prizes for standard chemicals and ^13^C-labeled acetone, material costs of €15–20 can be calculated to synthesize 10 mg of precursor **11** or **18**, which is the amount per liter minimal medium to achieve quantitative Trp-labeling. This is in sharp contrast to the current average prizes of commercial suppliers for isotope labeled early metabolic precursors like [1-^13^C]glucose (€240/g) [2-^13^C]glucose (€630/g), [4-^13^C]erythrose (€1100/g), [2-^13^C]pyruvate (€820/g), [3-^13^C]pyruvate (€1200/g) and [2-^13^C]glycerol (€520/g). Considering the high literature-reported concentrations required (1–3 g labeled compound/L medium) to achieve maximum incorporation levels, these cost issues severely affect their application for routine labeling of multiple protein samples (Teilum et al. 2006; Lundstrom et al. 2007; Kasinath et al. 2013; Weininger 2017; Milbradt et al. 2015).

To conclude, the novel tryptophan precursor anthranilic acid allows for heavy isotope incorporation into tryptophan side chains using *E. coli* protein overexpression with selectivities and incorporation levels, which were previously reserved only to cell-free protein synthesis. The economic advantages of our new labeling technique will prove beneficial for many future applications concerning the NMR-based studies of high molecular weight protein structure and dynamics.

## Electronic supplementary material

Below is the link to the electronic supplementary material.

Supplementary material 1 (PDF 1826 KB)
