# Supplementary material for: Anthranilic acid, the new player in the ensemble of aromatic residue labeling precursor compounds
Source: J Biomol NMR. 2017 Aug 31;69(1):13–22. doi: 10.1007/s10858-017-0129-2 (PMC5626795; doi:10.1007/s10858-017-0129-2)
Supplement: Supplementary file 1 — Supplementary material 1 (PDF 1826 KB) [file 10858_2017_129_MOESM1_ESM.pdf]

Supporting Information

1. Organic synthesis
   1.1 General comments
   1.2 Synthesis of [^{15}N]anthranilic acid **4**
   1.3 Synthesis of 3,5-dideuterio[4,6-$^{13}C_2$]anthranilic acid **11**
   1.4 Synthesis of 4,6-dideuterio[5-^{13}C]anthranilic acid **18**
2. NMR spectra of products and intermediates
3. Protein overexpression
4. Quantification of H6-GB1 labeling using [^{15}N]anthranilic acid **4**
5. NMR spectra of selectively Trp-^{13}C-labeled and ^{13}C-uniformly labeled Brd4-BD1
6. Literature

1. Organic Synthesis

1.1. General Methods: Unless otherwise stated, all reagents and reactants were purchased from commercial suppliers and used without further purification. All solvents were distilled prior to use. Dichloromethane was dried by elution over an aluminium oxide column and stored over molecular sieves (4 Å) and acetonitrile over molecular sieves (3 Å). D_2O (99.9% D) and DCl (7.6 N in D_2O) were purchased from Euriso-top®; urea ^{15}-N_2 (98% ^{15}N), $^{15}NH_4Cl$ (99% ^{15}N), [1,3-$^{13}C_2$]acetone (99% ^{13}C) and [2-^{13}C]acetone (99% ^{13}C) were purchased from ISOTEC Sigma Aldrich®. Oxygen- and moisture sensitive reactions were carried out under an argon atmosphere and yields refer to pure compounds. The reactions were monitored via thin layer chromatography (TLC) on silica gel 60 with fluorescent indicator UV254 by MACHEREY-NAGEL GmbH & Co. KG. Visualization of the compounds was carried out using an UV-lamp (254 nm) and by application of aqueous $KMnO_4$ (0.5%) solution with subsequent heating using a hot-gun. Flash column chromatography was performed on silica gel 60 (0.040-0.063 mm) from Merck. ^{1}H and ^{13}C NMR spectroscopic data for tryptophan precursors and intermediates were recorded on a Bruker AVANCE-DRX 400 MHz, 600 MHz or 700 MHz spectrometer. NMR solvent signals were calibrated to 4.79 ppm (D_2O) and 2.50 ppm (6d-DMSO). Chemical shifts (δ) are given in ppm (s = singlet, bs = broad singlet, d = doublet, dd = doublet of doublets, ddd = doublet of doublets of doublets, dddd = doublet of doublets of doublets of doublets, ddddd = doublet of doublets of doublets of doublets of doublets, qd = quartet of doublets, m = multiplet) and reported relative to the residual solvent peaks. Coupling constants (*J*) are given in Hertz (*Hz*). Protein NMR spectra were acquired at 298 K on an Agilent Inova 500 MHz spectrometer using a standard 5 mM triple resonance probehead. High resolution mass spectrometry experiments were performed using electrospray ionization (ESI, 3 keV, in the positive or negative ion mode). Continuous-flow hydrogenations were performed in an H-Cube® reactor from ThalesNano®. Microwave reactions were conducted in a Biotage Initiator® microwave synthesizer.

1.2. Synthesis of compound [^{15}N]anthranilic acid 4

[^{15}N]Phthalimide 3:[1] Phthalic anhydride (400 mg, 2.7 mmol) was heated to 250°C together with $^{15}NH_4Cl$ (177 mg, 3.3 mmol) and DMAP (33 mg, 0.3 mmol) using a microwave reactor. The microwave vessel was purged with argon and sealed before the reaction was started. After 50 min, the reaction mixture was allowed to cool to room temperature and diluted with acetone. The solids were filtered off and the filtrate was evaporated under

reduced pressure. The residual white solid was recrystallized from EtOH (5 mL) to get rid of surplus phthalic anhydride. Immediately after cooling white crystals appeared, which were filtered off, washed with cold EtOH and drying *in vacuo* yielded 266 mg of [^{15}N]phthalimide **3** (1.8 mmol, 66%) as white crystals.

Alternatively, product **3** was prepared according to literature.[2] A mixture of phthalic anhydride (2.200 g, 14.8 mmol) and [$^{15}N_2$]urea (300 mg, 4.8 mmol) in xylenes isomeric mixture (10 mL) was heated to 140°C for 4 h. During heating, white precipitate was formed and proceeded rapidly after cooling to room temperature. The precipitated solid was filtered off, washed with xylenes and recrystallized from EtOH (20 mL) to get rid of surplus phthalic anhydride. Immediately after cooling white crystals appeared, which were filtered off, washed with cold EtOH and drying *in vacuo* gave 1.203 g of [^{15}N]phthalimide **15** (8.1 mmol, 84%). ^{1}H-NMR (6d-DMSO, 600.25 MHz): δ=7.83 (s, 4H, C$H_{arom.}$), 11.33 (d, J = 93.88 Hz, 1H, ^{15}N*H*). ^{13}C-NMR (6d-DMSO, 100.66 MHz): δ=123.4, 133.1, 134.8, 169.7. ^{15}N-NMR (6d-DMSO, 60.82 MHz): δ=158.6. ESI-MS (pos. mode): calcd. for $C_8H_5{}^{15}NNaO_2$ $[M + Na]^+$ 171.0188; found 171.0186.

[^{15}N]Anthranilic acid 4:[2] To a solution of aqueous NaOH (20%, 17 mL) was gradually added Br_2 (296 µL, 5.8 mmol) while stirring at room temperature. The clear yellow reaction mixture was cooled to 0 °C and finely powdered [^{15}N]phthalimide **3** (856 mg, 5.8 mmol) was added in small portions via a spatula. The reaction mixture was stirred until everything was dissolved and then cooled to -5 °C. NaOH (609 mg, 15.2 mmol) was added and the solution was stirred vigorously for 30 min remaining at -5 °C. Subsequently, it was heated to 70 °C for 5 minutes and then aqueous $NaHSO_4$ (36%, 211 µL) was added. The hot and clear red solution was allowed to cool to room temperature and brought to pH > 7 by slow addition of conc. HCl at 0 °C. Careful addition of glacial acetic acid resulted in precipitation of [^{15}N]anthranilic acid **4**. The precipitate was filtered off, washed with cold water and drying *in vacuo* gave 423 mg of [^{15}N]anthranilic acid **4** (3.1 mmol, 53%) as a bright brown solid. ^{1}H-NMR (6d-DMSO, 600.25 MHz): δ=6.49 (ddd, J = 7.98Hz, 6.96 Hz, 1.02 Hz, 1H, C$H_{arom.}$), 6.72 (d, J = 8.40 Hz, 1H, C$H_{arom.}$), 7.21 (ddd, J = 8.37 Hz, 6.93 Hz, 1.53 Hz, 1H, C$H_{arom.}$), 7.67 (dd, J = 8.01 Hz, 1.47 Hz, 1H, C$H_{arom.}$). ^{13}C-NMR (6d-DMSO, 150.95 MHz): δ=110.0, 115.0, 116.8, 131.6, 134.2, 152.0, 170.0. ^{15}N-NMR (6d-DMSO, 60.83 MHz): δ=69.4. ESI-MS (neg. mode): calcd. for $C_7H_6{}^{15}NO_2$ $[M - H]^-$ 137.0374; found 137.0369.

1.2 Synthesis of 3,5-dideuterio[4,6-$^{13}C_2$]anthranilic acid 11

[5,7-$^{13}C_2$]3-Ethyl-4-hydroxyquinolin-2(1*H*)-one 7:[3,4] For the synthesis of [5,7-$^{13}C_2$] 3-ethyl-4-hydroxyquinolin-2(1*H*)-one **7**, [1,3-$^{13}C_2$]acetone **5** was converted over three steps to [3,5-$^{13}C_2$]aniline **6** as reported previously.[4] [3,5-$^{13}C_2$]Aniline **6** (680 mg, 7.1 mmol) and diethyl ethylmalonate (2.01 mL; 10.7 mmol) were heated in a metal bath to 230 °C for 1 h and then to 270 °C for 3.5 h. The flask was equipped with a distillation head and a Liebig condenser and during 4.5 h of heating 2 eq. of ethanol were distilled. After cooling the flask for approximately 3 minutes, toluene (12 mL) was carefully added while stirring and subsequently 0.5 N NaOH (12 mL). Remaining solid was removed *via* filtration and the two phases were separated. The aqueous phase was washed with toluene (3 x 20 mL) and then treated with 6 N HCl leading to the precipitation of an off-white solid, which was separated by filtration. Drying *in vacuo* yielded 1.05 g of [5,7-$^{13}C_2$]3-ethyl-4-hydroxyquinolin-2(1*H*)-one **7** (5.5 mmol, 77%) as a white solid. ^{1}H-NMR (6d-DMSO, 600.25 MHz): δ=1.00 (t, J = 7.38 Hz, 3H, CH_3), 2.56 (q, J = 7.36 Hz, 2H, CH_2), 7.12 (dd, J = 6.96 Hz, 1H, C$H_{arom.}$), 7.23 (d, J = 7.92 Hz, 1H, C$H_{arom.}$), 7.42 (dddd, J = 160.02 Hz, 7.48 Hz, 1H, ^{13}C$H_{arom.}$), 7.86 (ddd, J = 161.38 Hz, 8.10 Hz, 1H, ^{13}C$H_{arom.}$), 10.05 (s, 1H, N*H*), 11.29 (s, 1H, O*H*). ^{13}C-NMR (6d-DMSO, 150.95 MHz): δ=123.0 (^{13}CH$_{arom.}$), 130.1 (^{13}CH$_{arom.}$). ESI-MS (pos. mode): calcd. for $C_9{}^{13}C_2H_{11}NNaO_2$ $[M + Na]^+$ 214.0755; found 214.0742.

[5,7-$^{13}C_2$]3-Ethyl-3-hydroxyquinoline-2,4(1*H*,3*H*)-dione 8:[3] [5,7-$^{13}C_2$]3-Ethyl-4-hydroxyquinolin-2(1*H*)-one **7** (662 mg, 3.5 mmol) was treated with 0.5 N NaOH until everything was dissolved (~12 mL). To the solution was added dropwise over 1 h peroxyacetic acid (3.52 mL, 36-40 wt. % in acetic acid) leading to precipitation of a white solid. The reaction mixture was stirred for an additional hour. Afterwards the white precipitate was filtered off and washed carefully with a small portion of 5% K_2CO_3 to remove unreacted starting material. The solid was dried *in vacuo* giving 356 mg of pure [5,7-$^{13}C_2$]3-ethyl-3-hydroxyquinoline-2,4(1*H*,3*H*)-dione **8** (1.7 mmol, 50%) as a white solid. The filtrate was evaporated under reduced pressure to give additional crude [5,7-$^{13}C_2$]3-ethyl-3-hydroxyquinoline-2,4(1*H*,3*H*)-dione **8**, which was used without any further purification in the next step. ^{1}H-NMR (6d-DMSO, 600.25 MHz): δ=0.77 (t, *J* = 7.41 Hz, 3H, C*H$_3$*), 1.66-1.79 (m, 2H, C*H$_2$*), 5.66 (bs, 1H, O*H*), 7.06 (d, *J* = 7.98 Hz, 1H, C*H$_{arom.}$*), 7.10 (dd, *J* = 7.08 Hz, 1H, C*H$_{arom.}$*), 7.58 (dddd, *J* = 161.44 Hz, 7.80 Hz, 1H, ^{13}C*H$_{arom.}$*), 7.71 (ddd, *J* = 162.52 Hz, 7.83 Hz, 1H, ^{13}C*H$_{arom.}$*), 10.78 (bs, 1H, N*H*). ^{13}C-NMR (6d-DMSO, 150.95 MHz): δ=127.2 (^{13}CH$_{arom.}$), 136.4 (^{13}CH$_{arom.}$). ESI-MS (pos. mode): calcd. for $C_9{}^{13}C_2H_{11}NNaO_3$ [*M* + *Na*]$^+$ 230.0704; found 230.0702.

[4,6-$^{13}C_2$]2-(2-Oxobutanamido)benzoic acid 9:[3] The pure [5,7-$^{13}C_2$]3-ethyl-3-hydroxyquinoline-2,4(1*H*,3*H*)-dione **8** (350 mg, 1.7 mmol) was dissolved in ethanol (60%, 8.8 mL) and carefully treated with H_5IO_6 (34%, 7.5 mL). The mixture with a white precipitate was stirred at room temperature overnight. Next day the mixture was cooled for approximately 4 h in the fridge for further precipitation and afterwards the white precipitate was filtered off. The solid was washed with water and dried *in vacuo* yielding 351 mg of [4,6-$^{13}C_2$]2-(2-oxobutanamido)benzoic acid **9** (1.6 mmol, 93%) as white solid. The evaporated filtrate of crude [5,7-$^{13}C_2$]3-ethyl-3-hydroxyquinoline-2,4(1*H*,3*H*)-dione **8** was dissolved in ethanol (60%, 8.8 mL) and carefully treated with H_5IO_6 (7.5 mL). The mixture with a white precipitate was stirred at room temperature overnight. Next day the mixture was cooled for approximately 4 h in the fridge for further precipitation and afterwards the white precipitate was filtered off. They solid was washed with water and dried *in vacuo* yielding 230 mg of [4,6-$^{13}C_2$]2-(2-oxobutanamido)benzoic acid **9** (1.0 mmol) as a white solid. The combined yield of [4,6-$^{13}C_2$]2-(2-oxobutanamido)benzoic acid **9** over two steps (**7** → **9**) was 75% (581 mg, 2.6 mmol). ^{1}H-NMR (6d-DMSO, 600.25MHz): δ=1.02 (t, *J* = 7.14 Hz, 3H, C*H$_3$*), 2.95 (q, *J* = 7.14 Hz, 2H, C*H$_2$*), 7.23 (dd, *J* = 6.87 Hz, 1H, C*H$_{arom.}$*), 7.67 (dddd, *J* = 161.74 Hz, 7.64 Hz, 1H, ^{13}C*H$_{arom.}$*), 8.04 (ddd, *J* = 163.55 Hz, 7.67 Hz, 1H, ^{13}C*H$_{arom.}$*), 8.67 (d, *J* = 8.28 Hz, 1H, C*H$_{arom.}$*), 12.33 (s, 1H, N*H*), 13.82 (bs, 1H, COO*H*). ^{13}C-NMR (6d-DMSO, 150.95 MHz): δ=132.0 (^{13}CH$_{arom.}$), 134.8 (^{13}CH$_{arom.}$). ESI-MS (pos. mode): calcd. for $C_9{}^{13}C_2H_{11}NNaO_4$ [*M* + *Na*]$^+$ 246.0653; found 246.0645.

[4,6-$^{13}C_2$]Anthranilic acid 10:[3] [4,6-$^{13}C_2$]2-(2-Oxobutanamido)benzoic acid **9** (280 mg, 1.3 mmol) was diluted in water (4.6 mL) and HCl$_{conc.}$ (6.1 mL). The reaction mixture was refluxed at 120 °C for 4 h and afterwards the hot mixture was filtered. The filtrate was evaporated *in vacuo* giving 290 mg of [4,6-$^{13}C_2$]anthranilic acid hydrochloride as a yellow-white solid. ^{1}H-NMR (D_2O, 600.25 MHz): δ=7.30 (d, *J* = 7.92 Hz, 1H, C*H$_{arom.}$*), 7.38-7.41 (m, 1H, C*H$_{arom.}$*), 7.57 (ddddd, *J* = 163.08 Hz, 7.74 Hz, 1.56 Hz, 1H, ^{13}C*H$_{arom.}$*), 8.01 (dddd, *J* = 165.55 Hz, 7.79 Hz, 1.44 Hz, 1H, ^{13}C*H$_{arom.}$*). ^{13}C-NMR (D_2O, 150.95 MHz): δ=132.1 (^{13}CH$_{arom.}$), 134.6 (^{13}CH$_{arom.}$). ESI-MS (neg. mode): calcd. for $C_5{}^{13}C_2H_6NO_2$ [*M* - *H*]$^-$ 138.0471; found 138.0471. To remove surplus salt, the product was dissolved in water, brought to pH=3-4 with 1 N HCl and extracted with dichloromethane (5 x 10 mL). The organic phase was dried over $MgSO_4$ and evaporated under reduced pressure to give 159 mg of [4,6-$^{13}C_2$]anthranilic acid **10** (1.1 mmol, 91%) as an off-white solid.

3,5-Dideuterio[4,6-$^{13}C_2$]anthranilic acid 11: [4,6-$^{13}C_2$]Anthranilic acid **10** (200 mg, 1.1 mmol) was diluted in D_2O (4 mL) and treated with 4 drops of $HCl_{conc.}$ in a microwave vessel. After irradiation of the mixture for 40 minutes at 120 °C, the solvent was evaporated and the solid was dried *in vacuo* yielding the hydrochloride salt of 3,5-dideuterio [4,6-$^{13}C_2$]anthranilic acid **11** as an off-white solid. To remove surplus salt, the product was dissolved in water and acidified with 1 N HCl to pH = 3-4. The aqueous phase was extracted with dichloromethane (5 x 10 mL), the combined organic phases were dried over $MgSO_4$ and evaporated under reduced pressure to give 148 mg of 3,5-dideuterio[4,6-$^{13}C_2$]anthranilic acid **11** (1.0 mmol, 92%) as an off-white solid. ^{1}H-NMR (6d-DMSO, 600.25 MHz): δ=7.21 (ddd, J = 1.20 Hz, 7.08 Hz, 157.51 Hz, 1H, $^{13}CH_{arom.}$), 7.67 (ddd, J = 1.38 Hz, 8.31 Hz, 159.52 Hz, 1H, $^{13}CH_{arom.}$). ^{13}C-NMR (6d-DMSO, 150.95 MHz): δ=131.5 ($^{13}CH_{arom.}$), 134.0 ($^{13}CH_{arom.}$). ESI-MS (neg. mode): calcd. for $C_5{}^{13}C_2H_4D_2NO_2$ $[M - H]^-$ 142.0597; found 142.0596.

Alternatively, 3,5-dideuterio[4,6-$^{13}C_2$]anthranilic acid **11** was prepared via the following route: [4,6-$^{13}C_2$]2-(2-oxobutanamido)benzoic acid **9** (280 mg, 1.3 mmol) was diluted in DCl (7.6 N in D_2O, 6.0 mL) and refluxed at 120 °C for 4 h. Afterwards the hot mixture was filtered and evaporated under reduced pressure. The product was dissolved in water and acidified with 1 N HCl to pH = 3-4. The aqueous phase was extracted with dichloromethane (5 x 10 mL), the combined organic phases were dried over $MgSO_4$ and evaporated under reduced pressure to give 166 mg of 3,5-dideuterio[4,6-$^{13}C_2$]anthranilic acid **11** (1.2 mmol, 94%) as an off-white solid.

1.3. Synthesis of 4,6-dideuterio[5-^{13}C]anthranilic acid 18

5,7,8-Trideuterio[6-^{13}C]3-ethyl-4-hydroxyquinolin-2(1*H*)-one 15:[3,4] For the synthesis of 5,7,8-trideuterio[6-^{13}C]3-ethyl-4-hydroxyquinolin-2(1*H*)-one **15**, [2-^{13}C]acetone **12** was converted over five steps to 2,3,5,6-tetradeuterio[4-^{13}C]aniline as reported previously.[4] The synthesis of compound **15** was performed as described for compound **7**, but using 259 mg (2.6 mmol) of 2,3,5,6-tetradeuterio[4-^{13}C]aniline as a substrate. The reaction gave 352 mg of compound **15** (69%, 1.8 mmol) as an off-white solid. ^{1}H-NMR (6d-DMSO, 700.40 MHz): δ=1.01 (t, J = 7.28 Hz, 3H, CH_3), 2.56 (q, J = 7.17 Hz, 2H, CH_2), 7.12 (d, J = 161.79 Hz, 1H, $^{13}CH_{arom.}$), 10.01 (s, 1H, N*H*), 11.26 (s, 1H, O*H*). ^{13}C-NMR (6d-DMSO, 176.13 MHz): δ=121.1 ($^{13}CH_{arom.}$). ESI-MS (pos. mode): calcd. for $C_{10}{}^{13}CH_8D_3NNaO_2$ $[M + Na]^+$ 216.0909; found 216.0904.

5,7,8-Trideuterio[6-^{13}C]3-ethyl-3-hydroxyquinoline-2,4(1*H*,3*H*)-dione 16:[3] The reaction was accomplished as described for the synthesis of compound **8**, but using 5,7,8-trideuterio[6-^{13}C]3-ethyl-4-hydroxyquinolin-2(1*H*)-one **15** (310 mg, 1.6 mg) as a substrate. The reaction gave 151 mg of pure compound **16** (45%, 0.7 mmol) as well as crude compound **16**, which were both used without any further purification in the following step. ^{1}H-NMR (6d-DMSO, 600.25 MHz): δ=0.77 (t, J = 7.38 Hz, 3H, CH_3), 1.67-1.75 (m, 2H, CH_2), 7.10 (d, J = 164.23 Hz, 1H, $^{13}CH_{arom.}$), 10.83 (bs, 1H, N*H*). ^{13}C-NMR (6d-DMSO, 150.95 MHz): δ=122.7 ($^{13}CH_{arom.}$). ESI-MS (pos. mode): calcd. for $C_{10}{}^{13}CH_8D_3NNaO_3$ $[M + Na]^+$ 232.0858; found 232.0851.

3,4,6-Trideuterio[5-^{13}C]2-(2-oxobutanamido)benzoic acid 17:[3] The product was prepared as described for compound **9** using pure (140 mg, 0.7 mmol) and crude compound **16** separately as substrates. Both reactions combined yielded 252 mg of 2-(2-oxobutanamido)benzoic-5-^{13}C-3,4,6-d_3 acid **17** (72%, 1.1 mmol) as a white solid over two steps. ^{1}H-NMR (6d-DMSO, 600.25MHz): δ=1.02 (t, J = 7.14 Hz, 3H, CH_3), 2.95 (q, J = 7.14 Hz, 2H, CH_2), 7.23 (d, J = 164.17 Hz, 1H, $^{13}CH_{arom.}$), 12.33 (s, 1H, N*H*), 13.82 (bs, 1H, COO*H*). ^{13}C-NMR (6d-DMSO, 150.95 MHz): δ=123.9 ($^{13}CH_{arom.}$). ESI-MS (neg. mode): calcd. for $C_{10}{}^{13}CH_7D_3NO_4$ $[M - H]^-$ 224.0837; found 224.0837.

4,6-Dideuterio[5-^{13}C]anthranilic acid 18: 3,4,6-Trideuterio[5-^{13}C]2-(2-oxobutanamido)benzoic acid **17** (240 mg, 1.1 mmol) was diluted in water (3.9 mL) and $HCl_{conc.}$ (5.2 mL). The reaction mixture was refluxed at 120 °C for 4 h and afterwards the hot mixture was filtered. The filtrate was evaporated *in vacuo*, dissolved in water, brought to pH=3-4 with 1 N HCl and extracted with dichloromethane (5 x 10 mL). The combined organic phases were dried over $MgSO_4$ and evaporated under reduced pressure to give 134 mg of 4,6-dideuterio[5-^{13}C]anthranilic acid **18** (1.0 mmol, 90%) as an off-white solid. ^{1}H-NMR (6d-DMSO, 600.25 MHz): δ=6.49 (d, *J* = 162.61 Hz, 1H, $^{13}CH_{arom.}$), 6.72 (d, *J* = 8.04 Hz, 1H, $CH_{arom.}$). ^{13}C-NMR (6d-DMSO, 150.95 MHz): δ=114.8 ($^{13}CH_{arom.}$). ESI-MS (neg. mode): calcd. for $C_6{}^{13}CH_4D_2NO_2$ $[M - H]^-$ 139.0563; found 139.0564.

2. NMR spectra of products and intermediates

[^{15}N]Phthalimide **3**

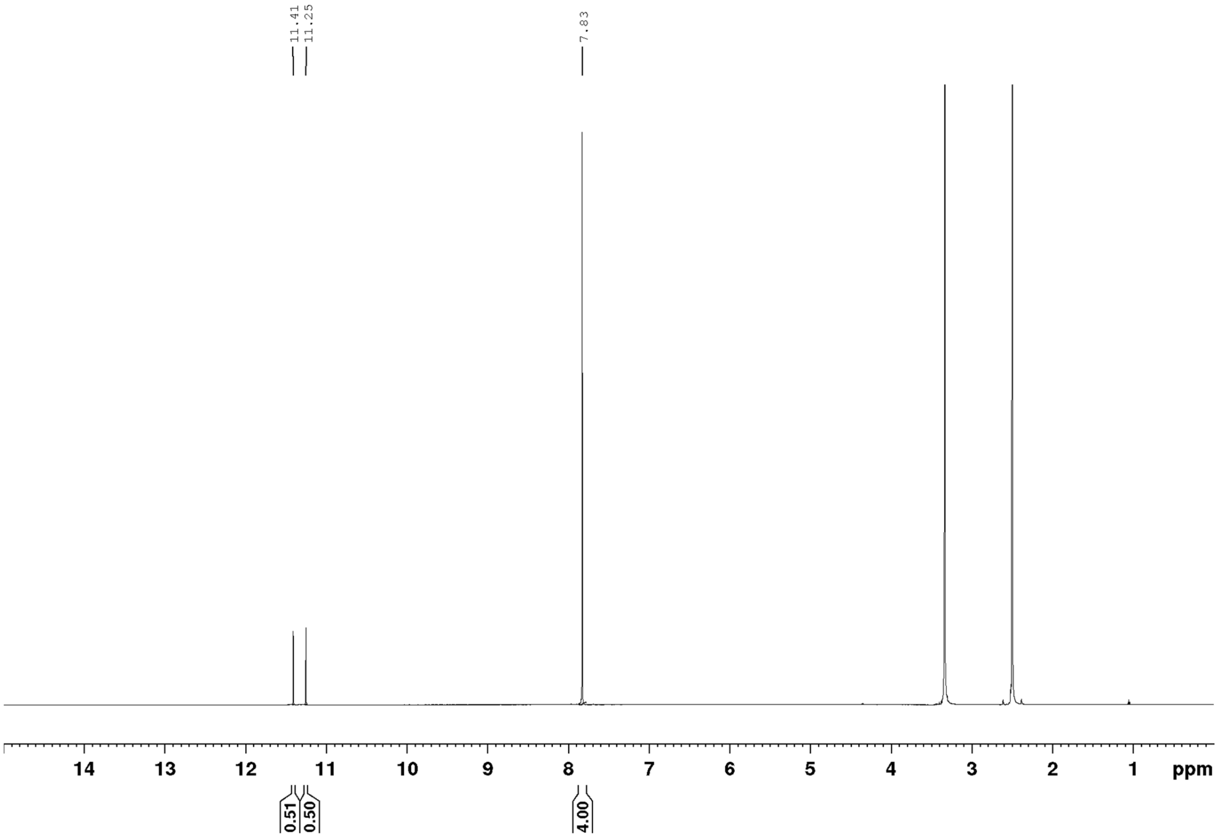

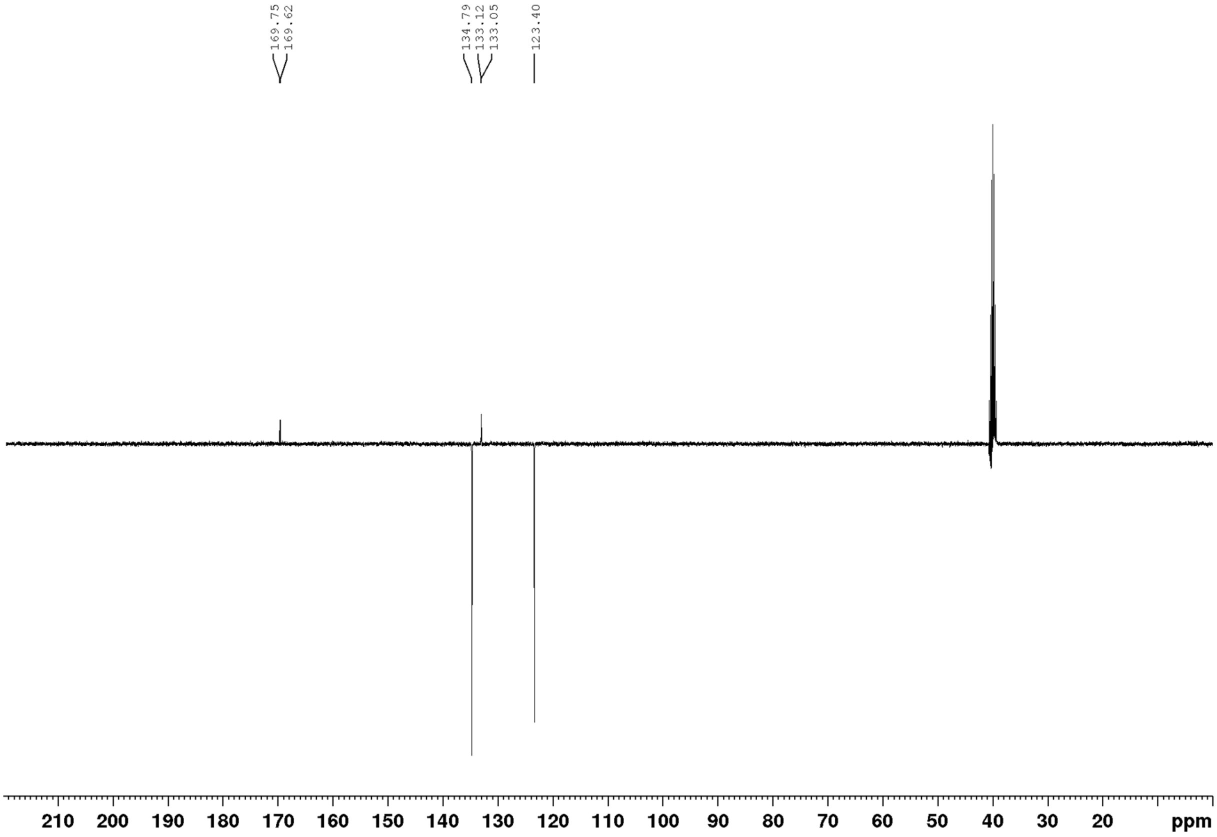

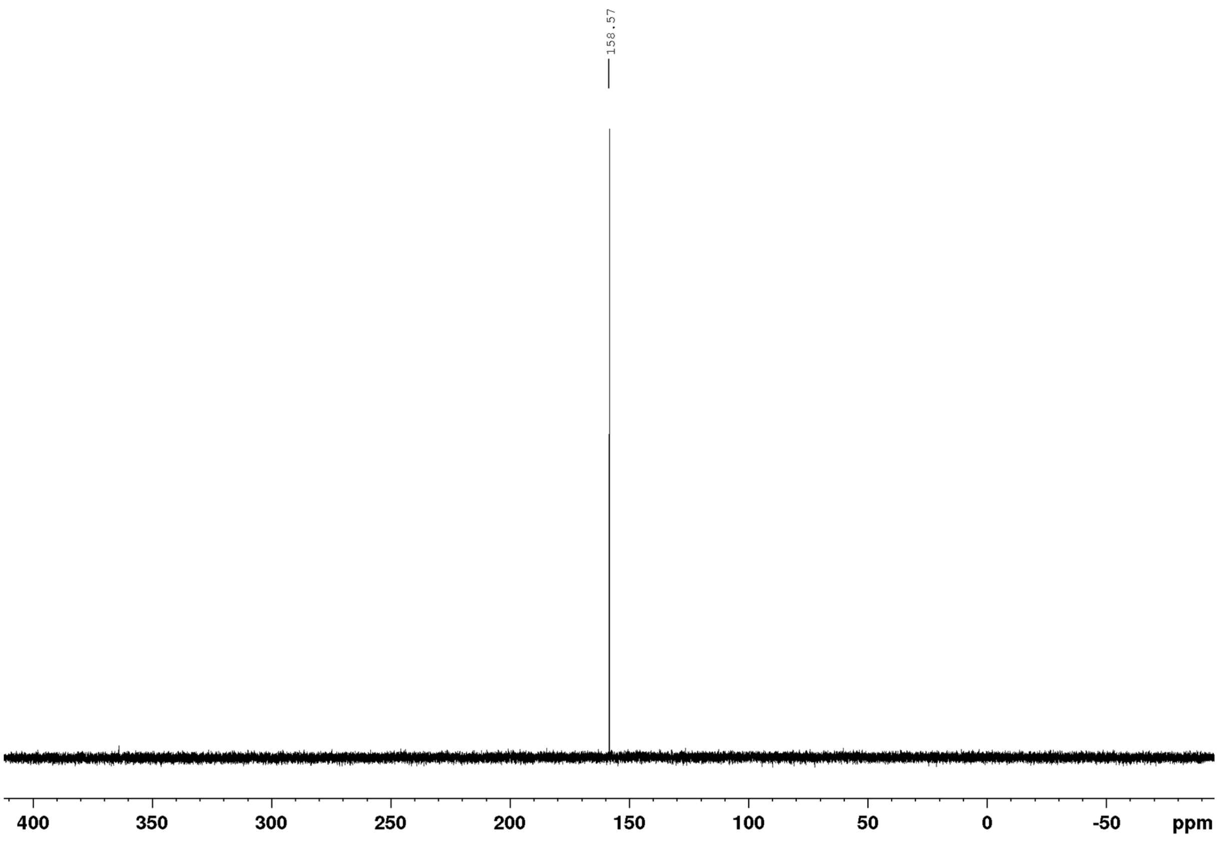
158.57
400
350
300
250
200
150
100
50
0
-50
ppm

[^{15}N]Anthranilic acid **4**

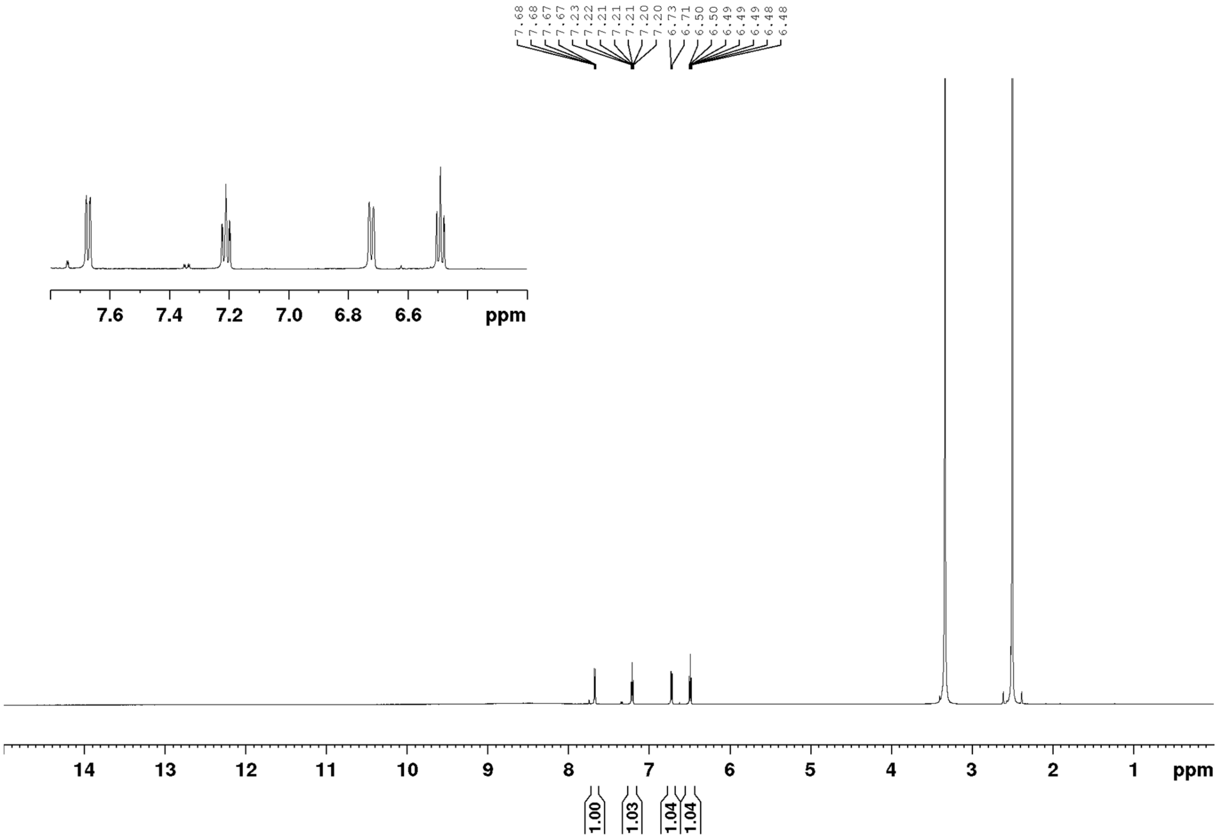

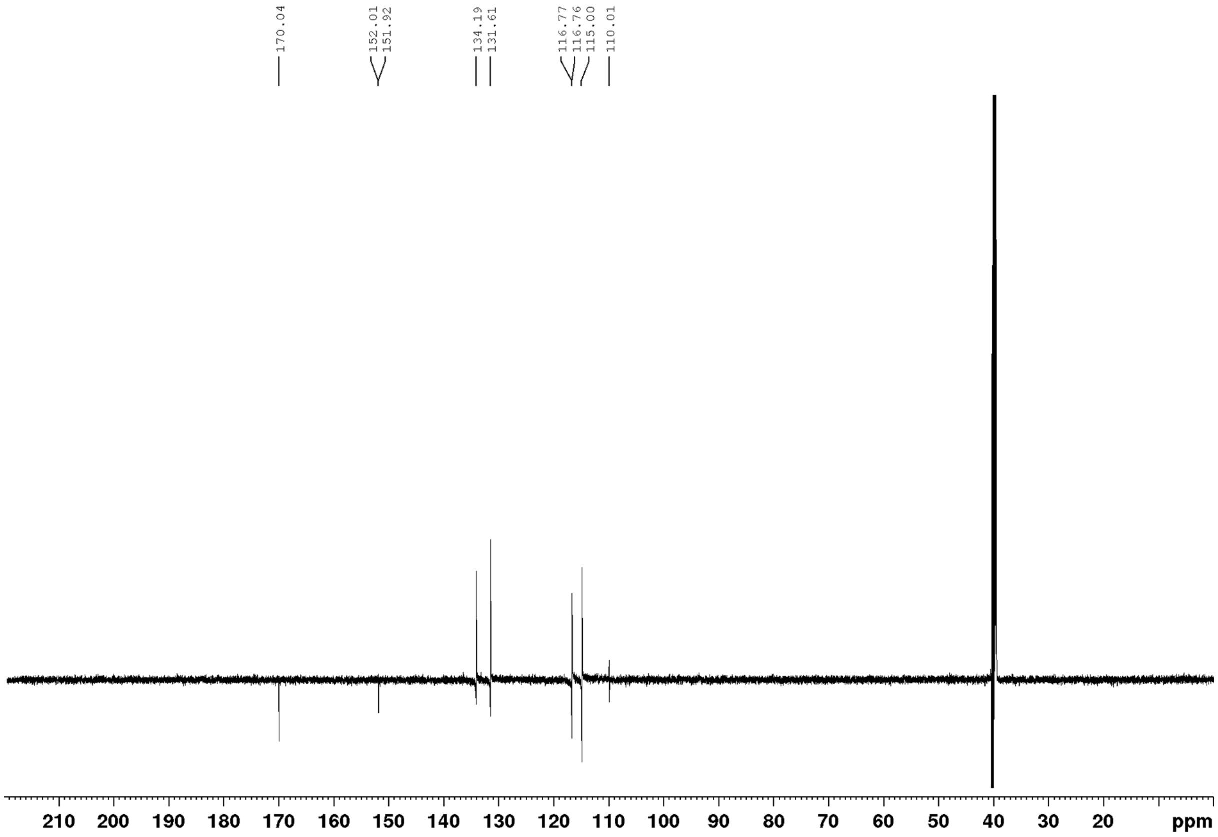

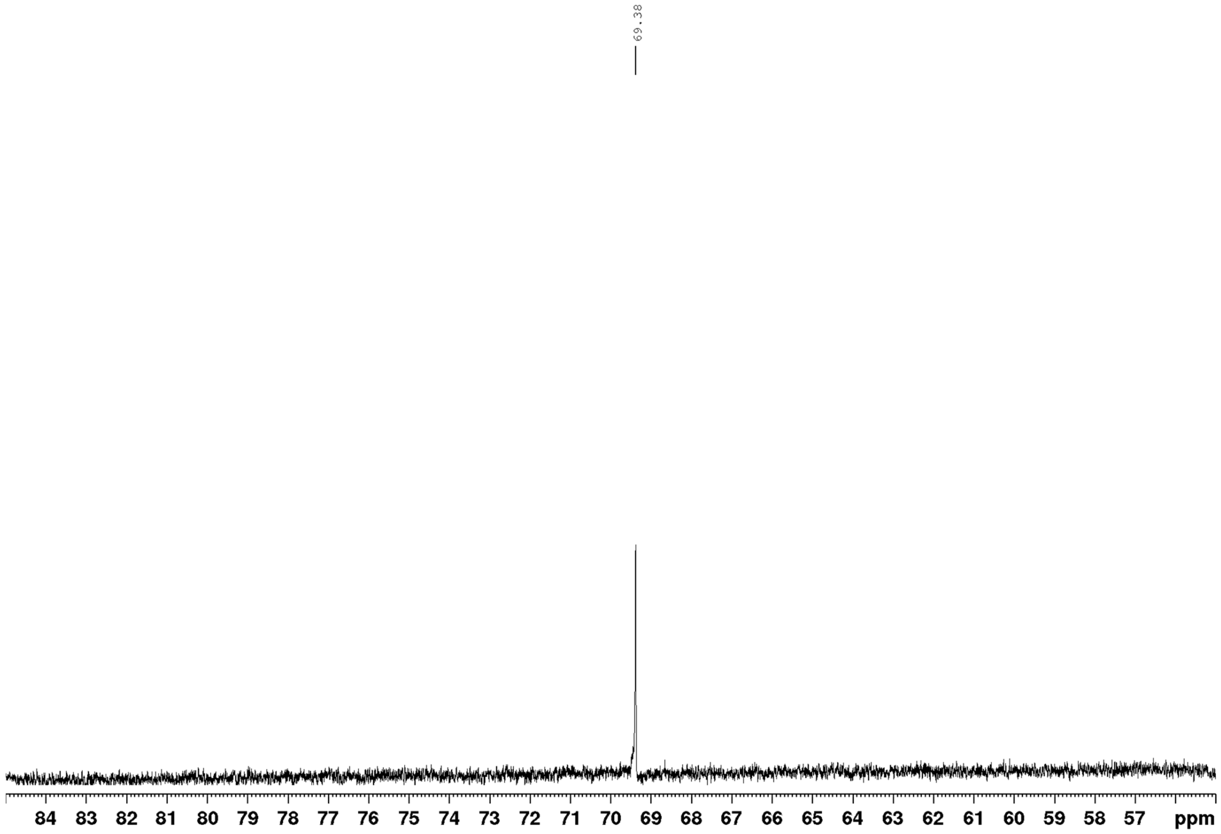
69.38
84 83 82 81 80 79 78 77 76 75 74 73 72 71 70 69 68 67 66 65 64 63 62 61 60 59 58 57 ppm

[5,7-$^{13}C_2$]3-Ethyl-4-hydroxyquinolin-2(1*H*)-one **7**

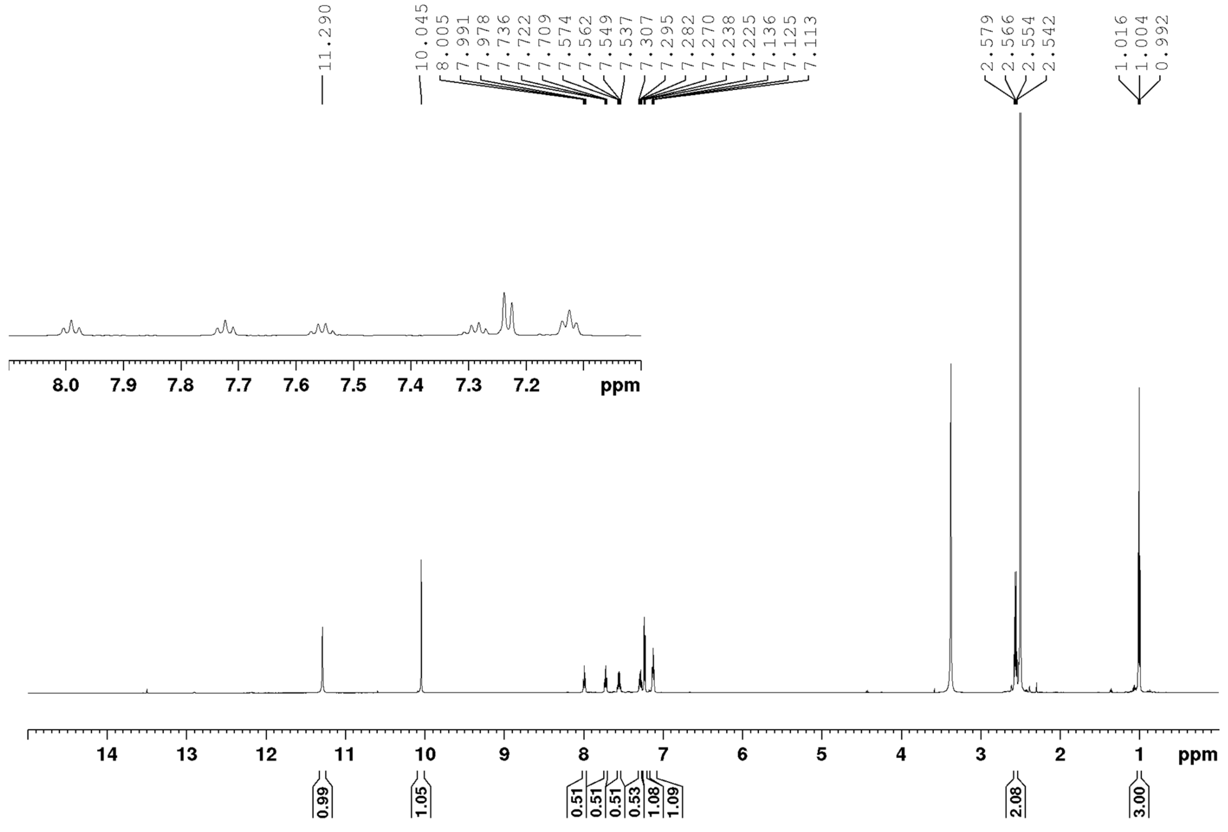

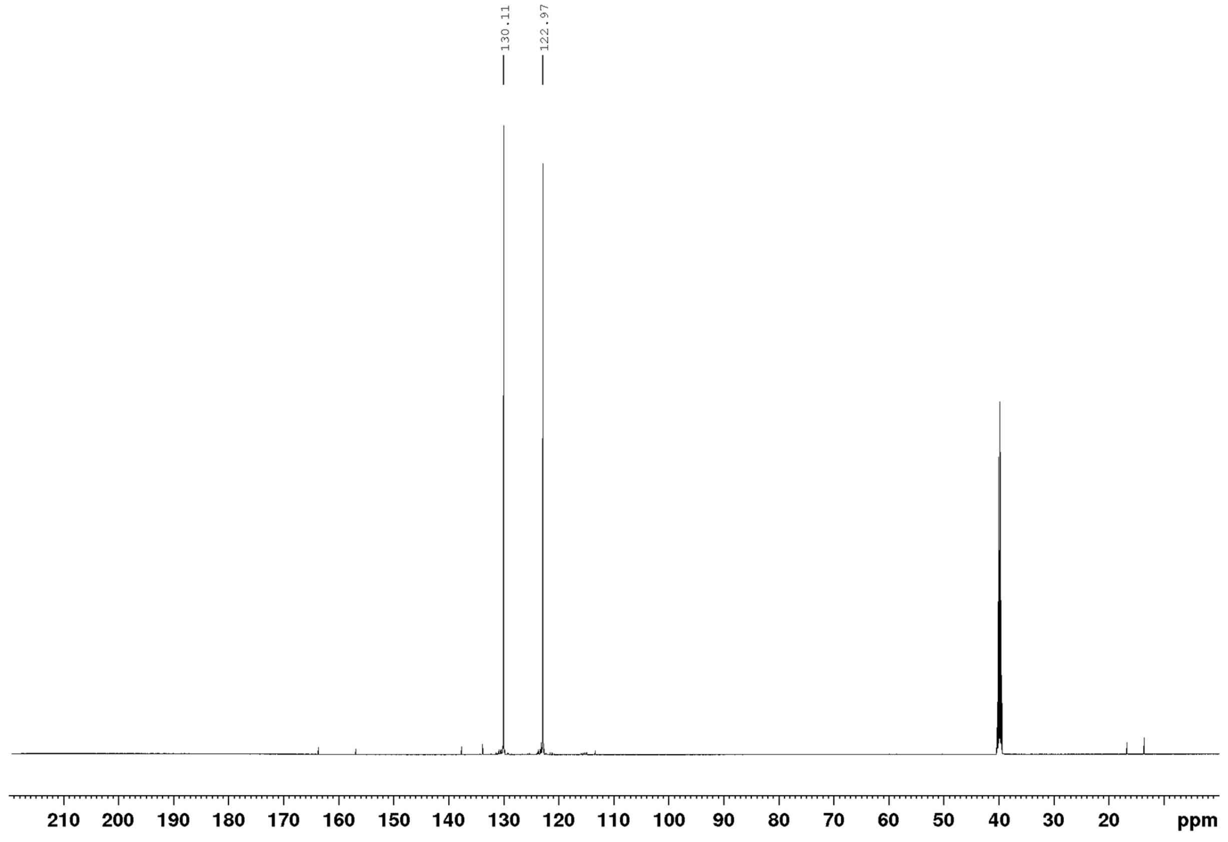

[5,7-$^{13}C_2$]3-Ethyl-3-hydroxyquinoline-2,4(1*H*,3*H*)-dione **8**

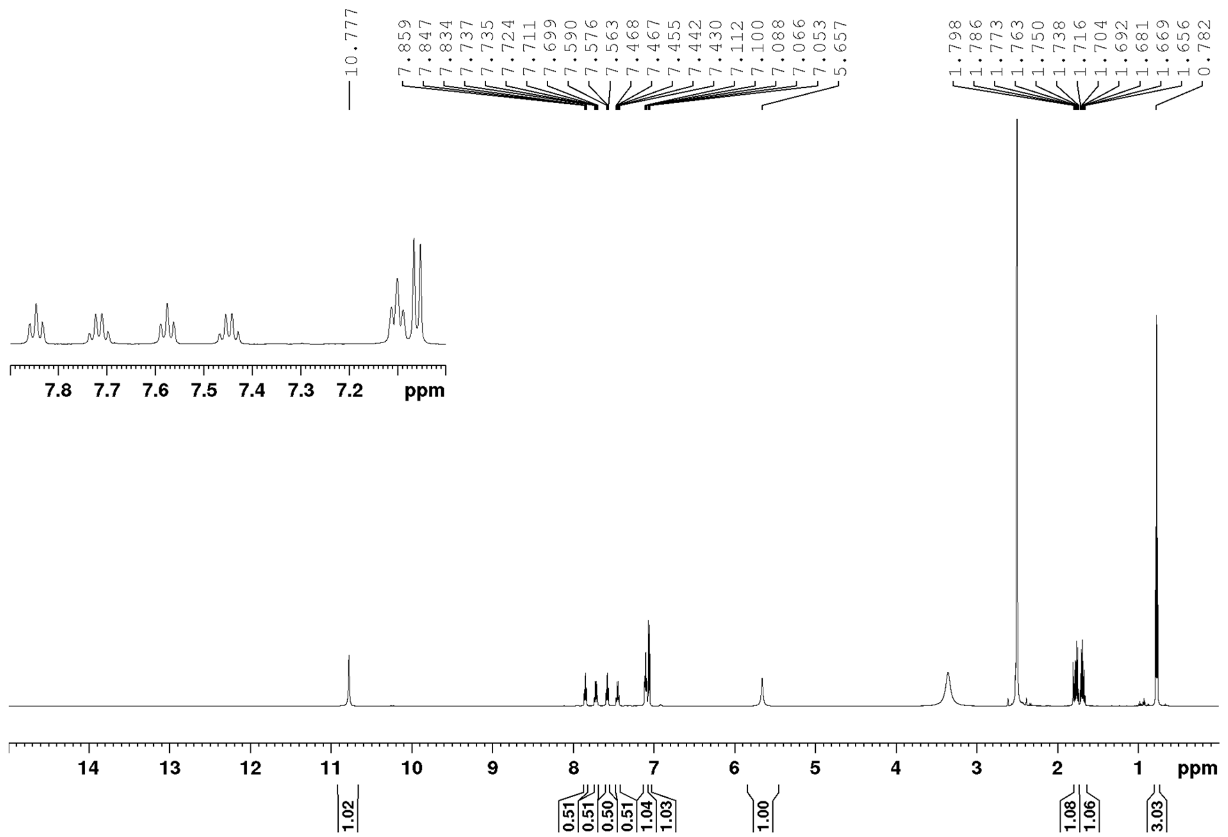

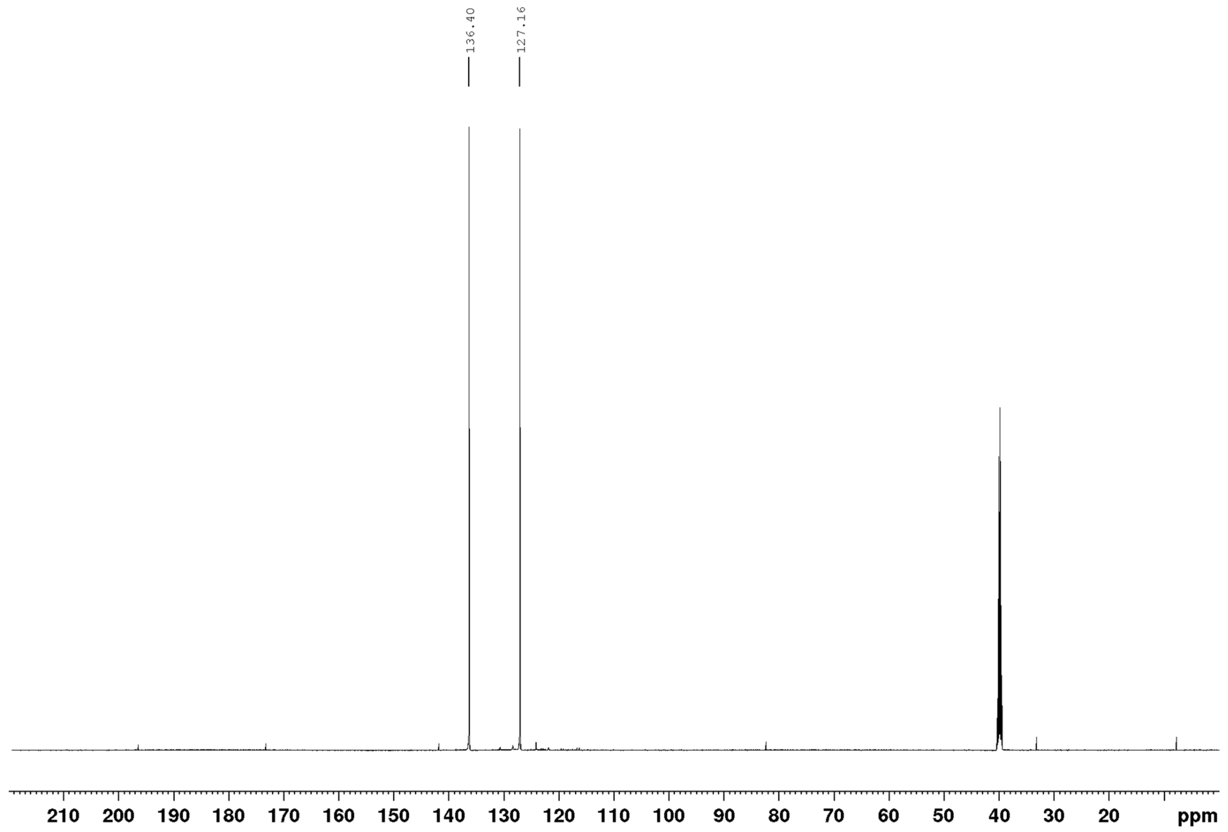

[4,6-$^{13}C_2$]2-(2-Oxobutanamido)benzoic acid **9**

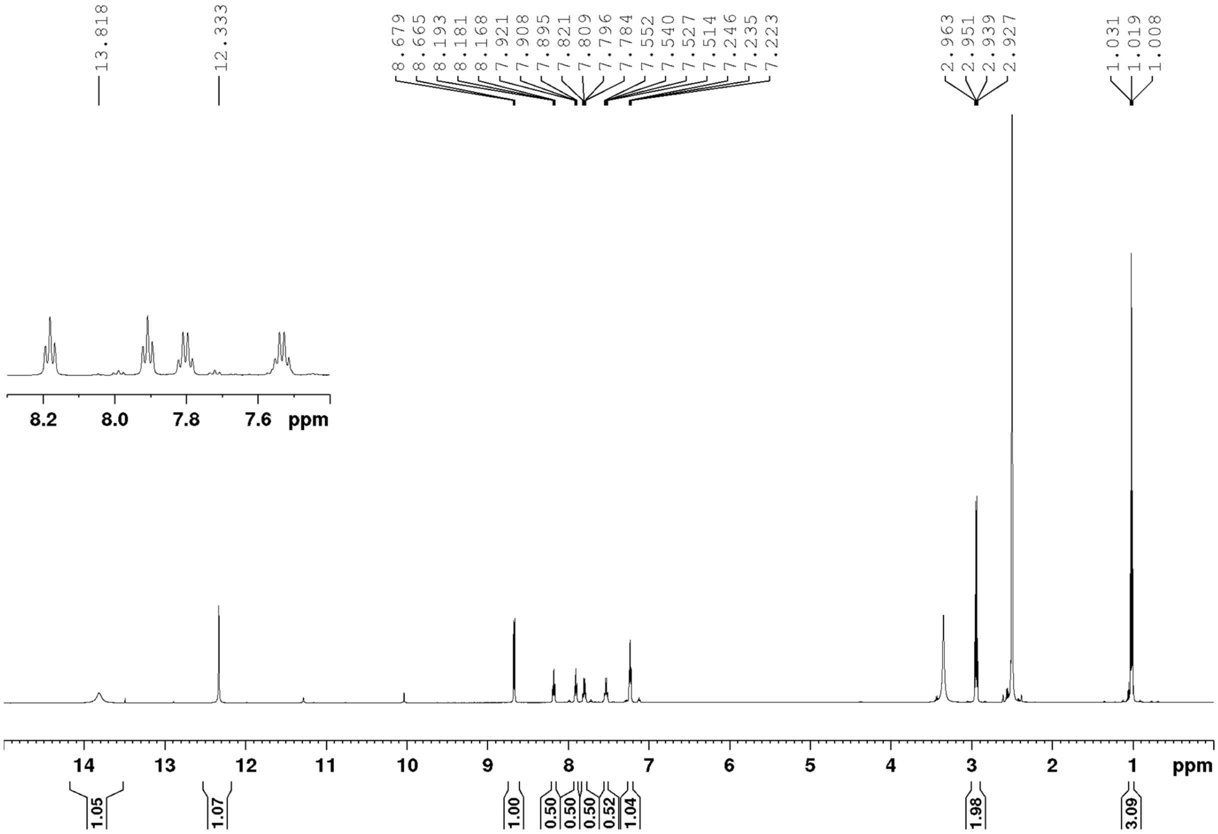

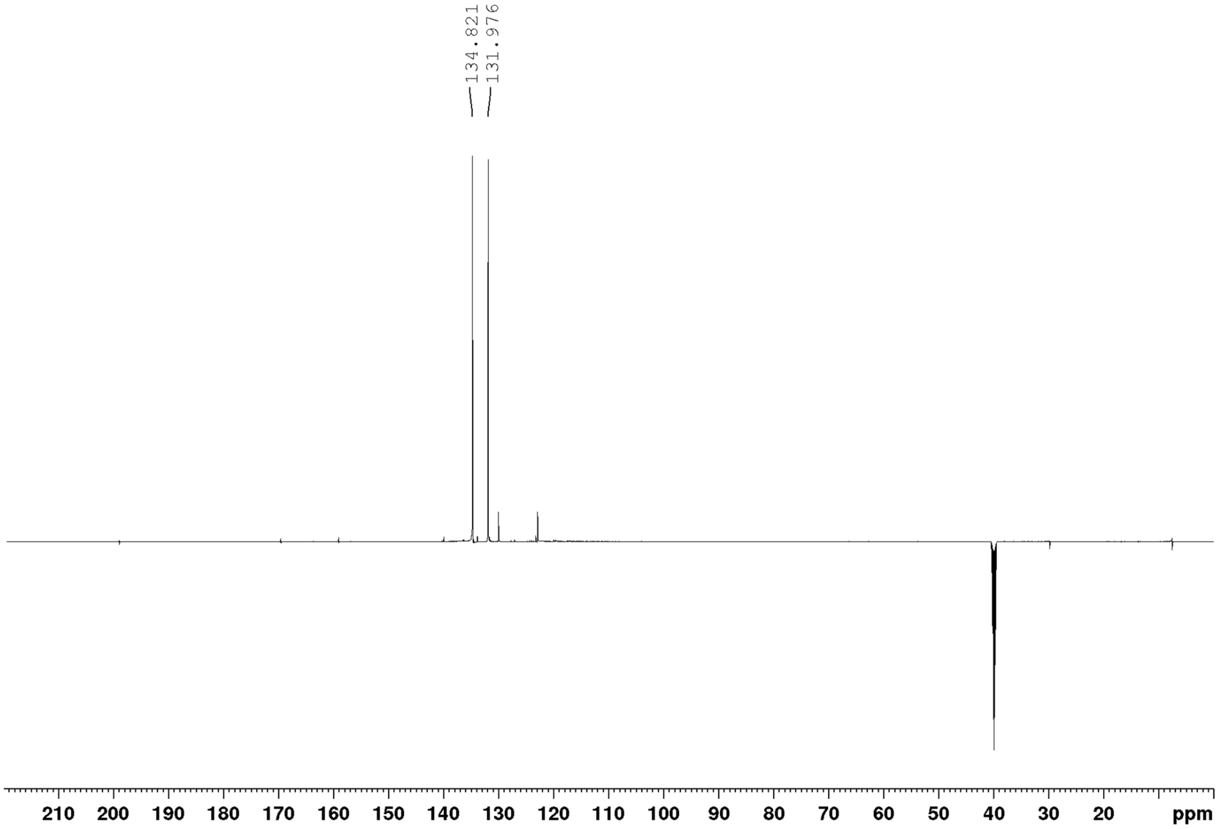

[4,6-$^{13}C_2$]Anthranilic acid hydrochloride **10**

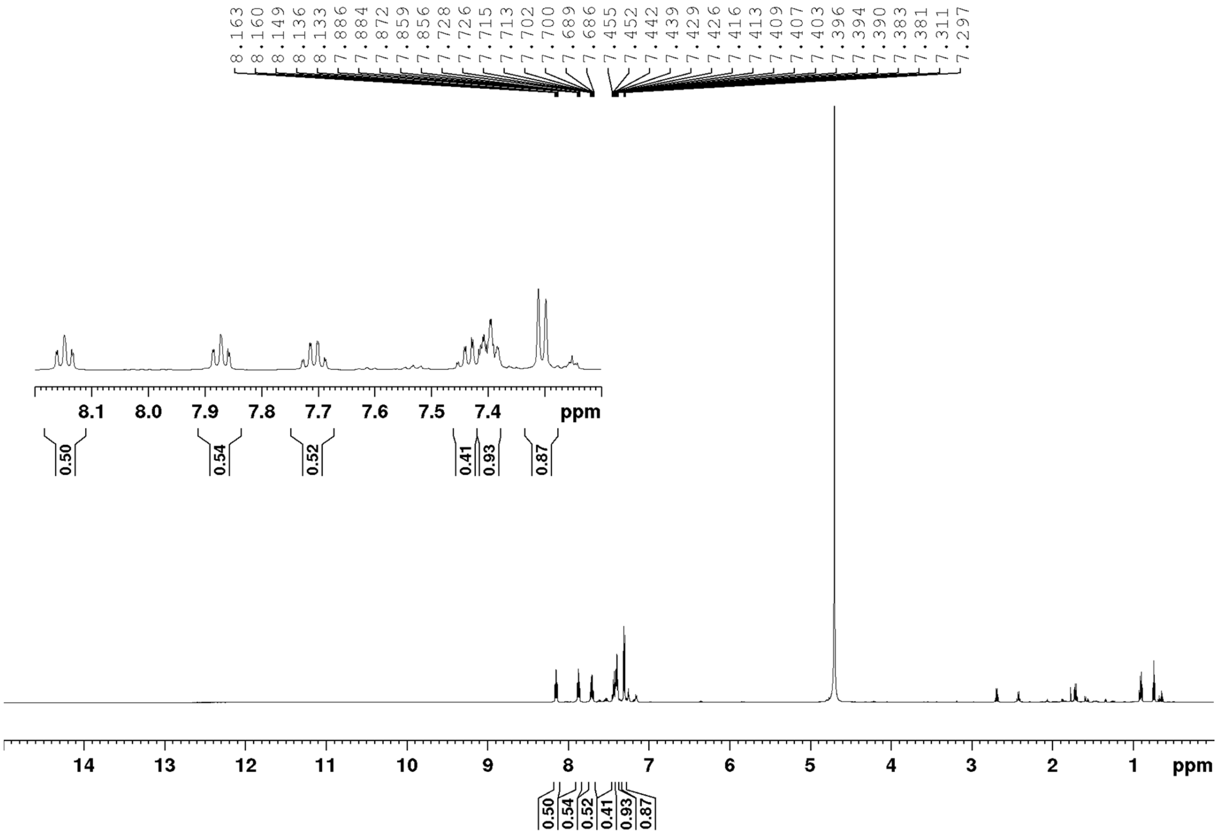

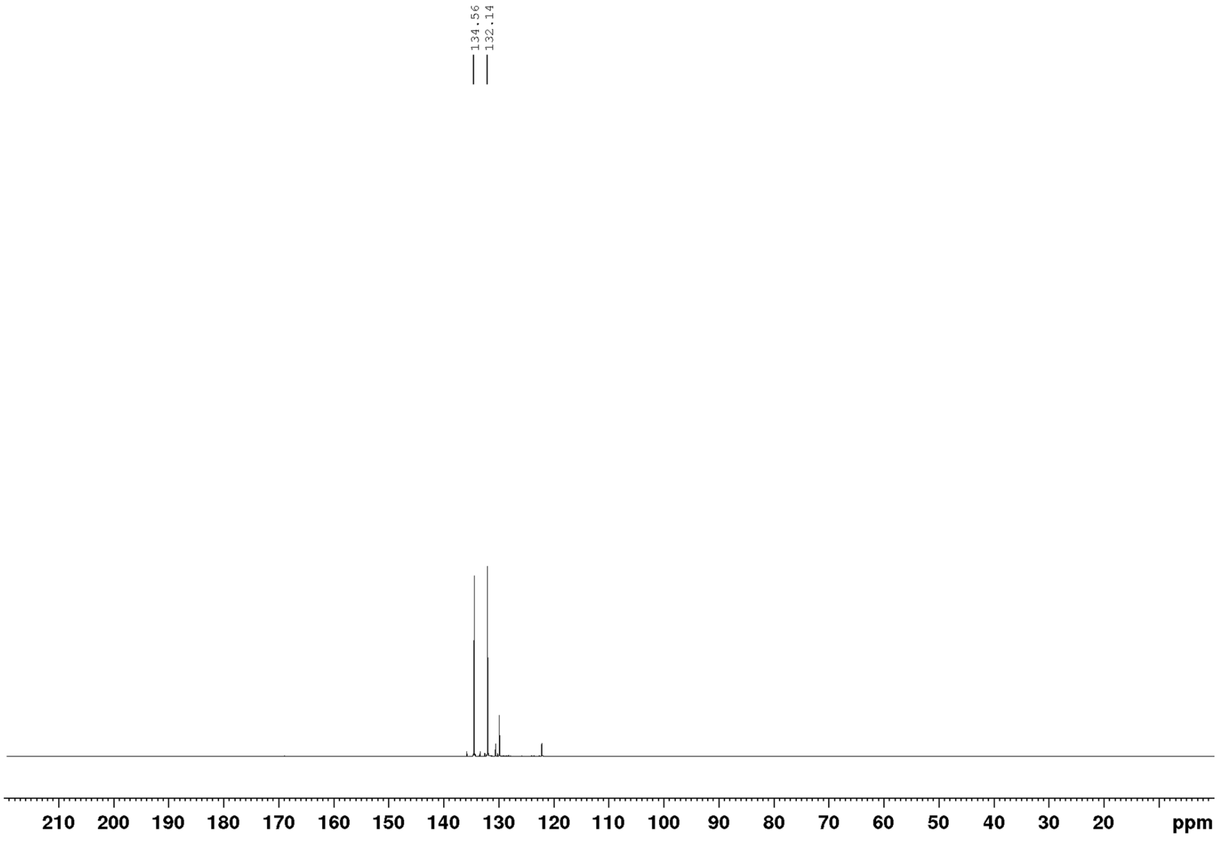

3,5-Dideuterio[4,6-$^{13}C_2$]anthranilic acid **11**

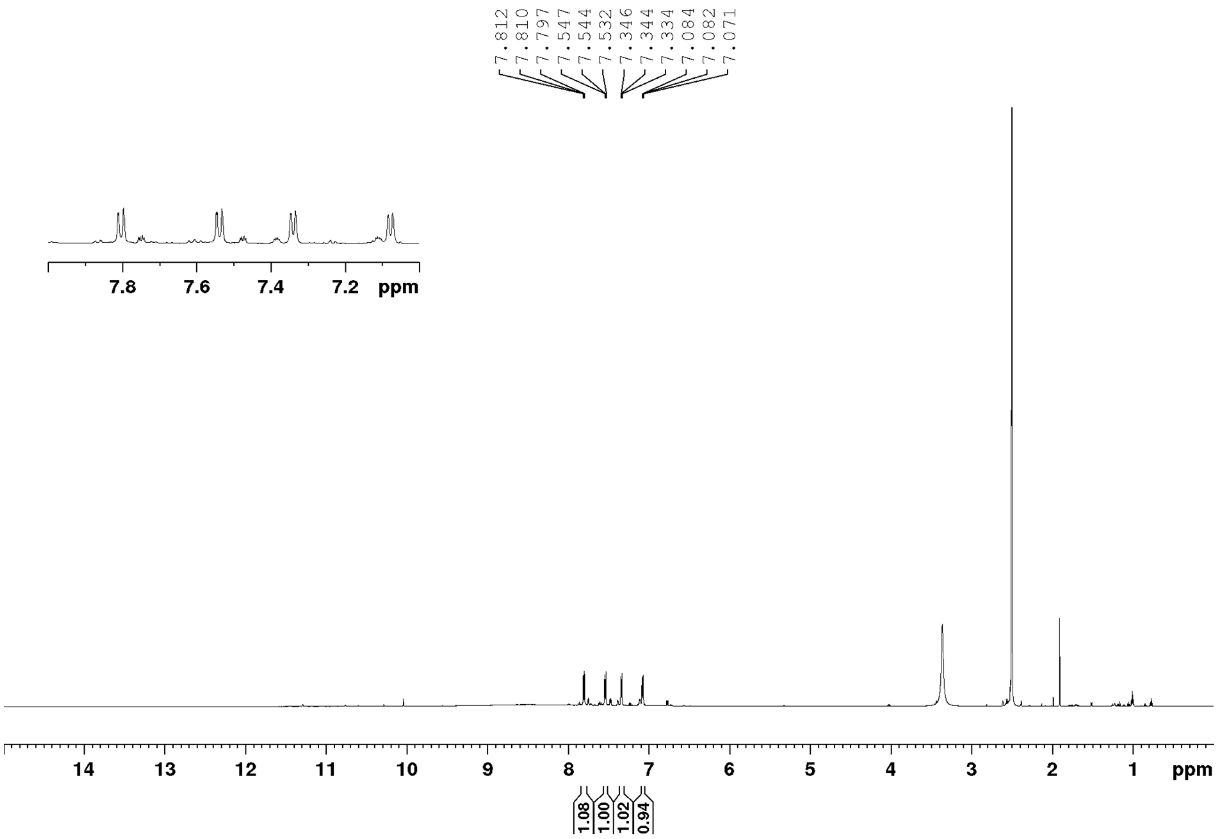

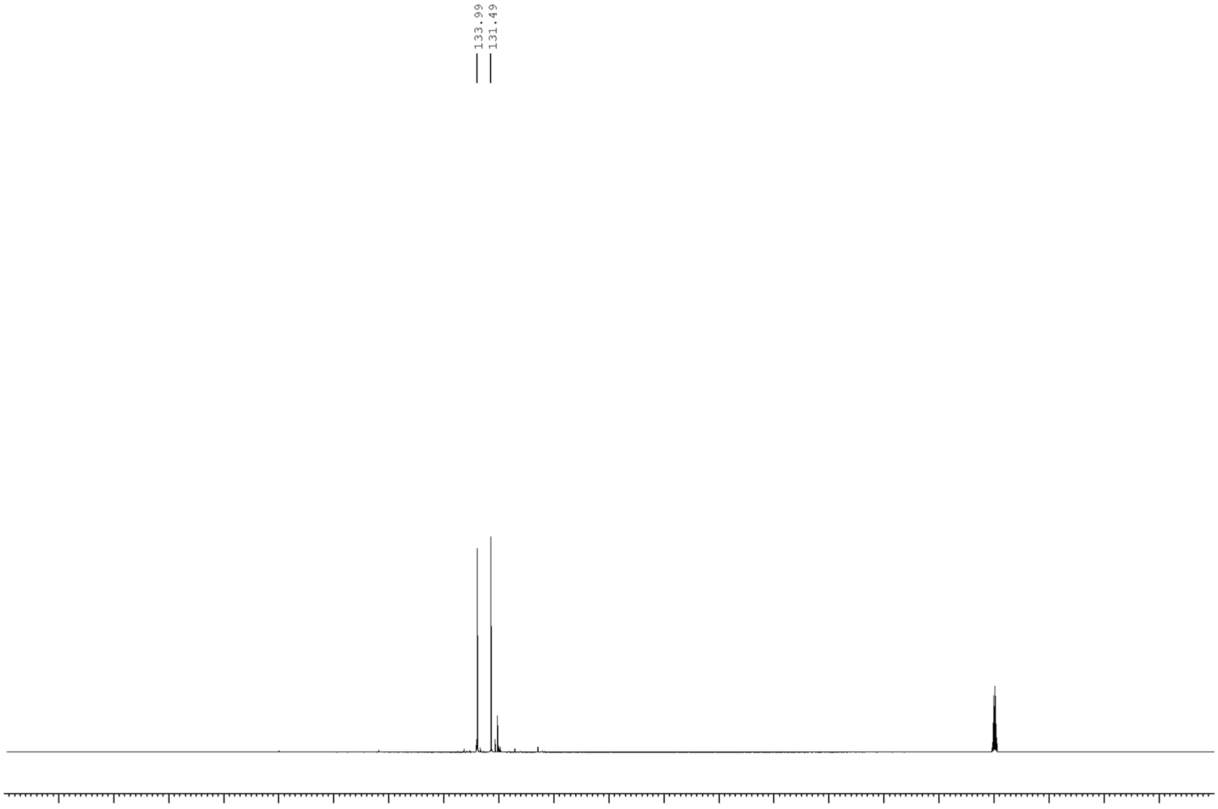

5,7,8-Trideuterio[6-^{13}C]3-ethyl-4-hydroxyquinolin-2(1*H*)-one **15**

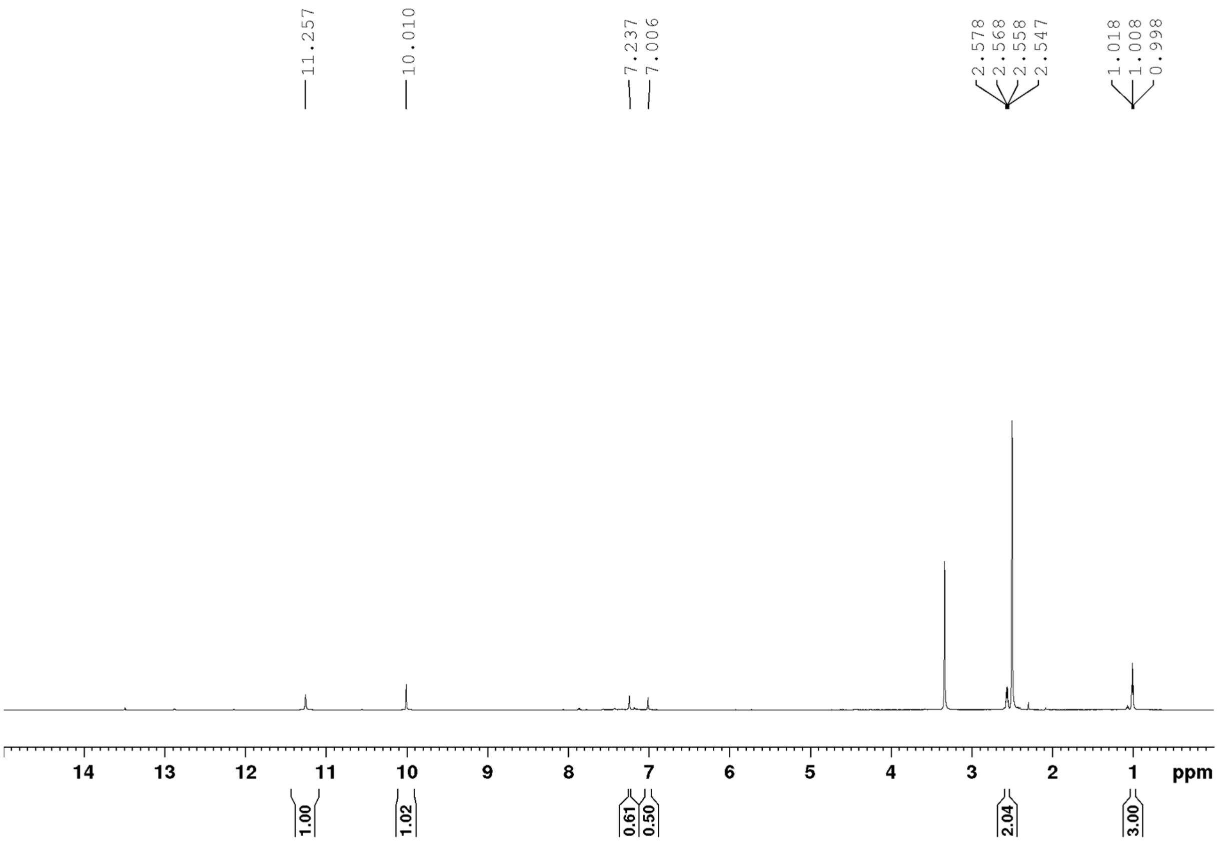

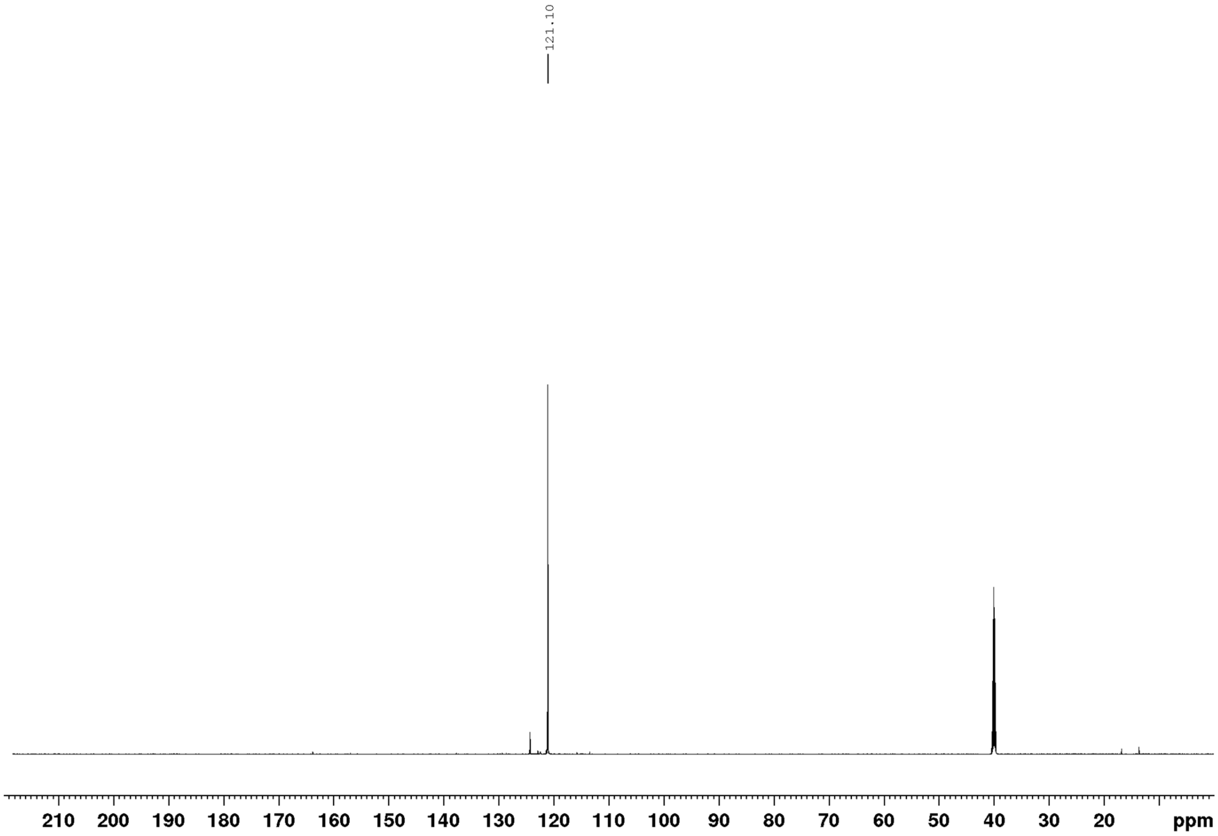

5,7,8-Trideuterio[6-^{13}C]3-ethyl-3-hydroxyquinoline-2,4(1*H*,3*H*)-dione **16**

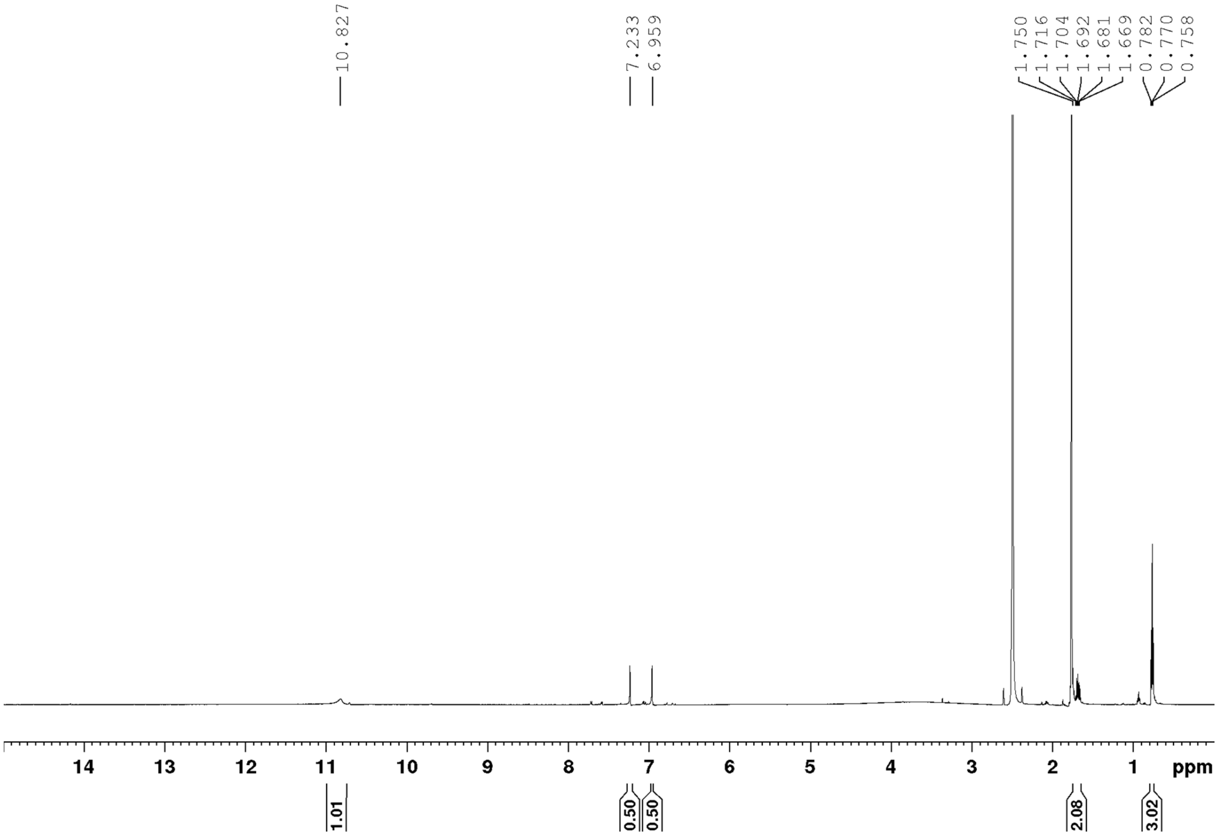

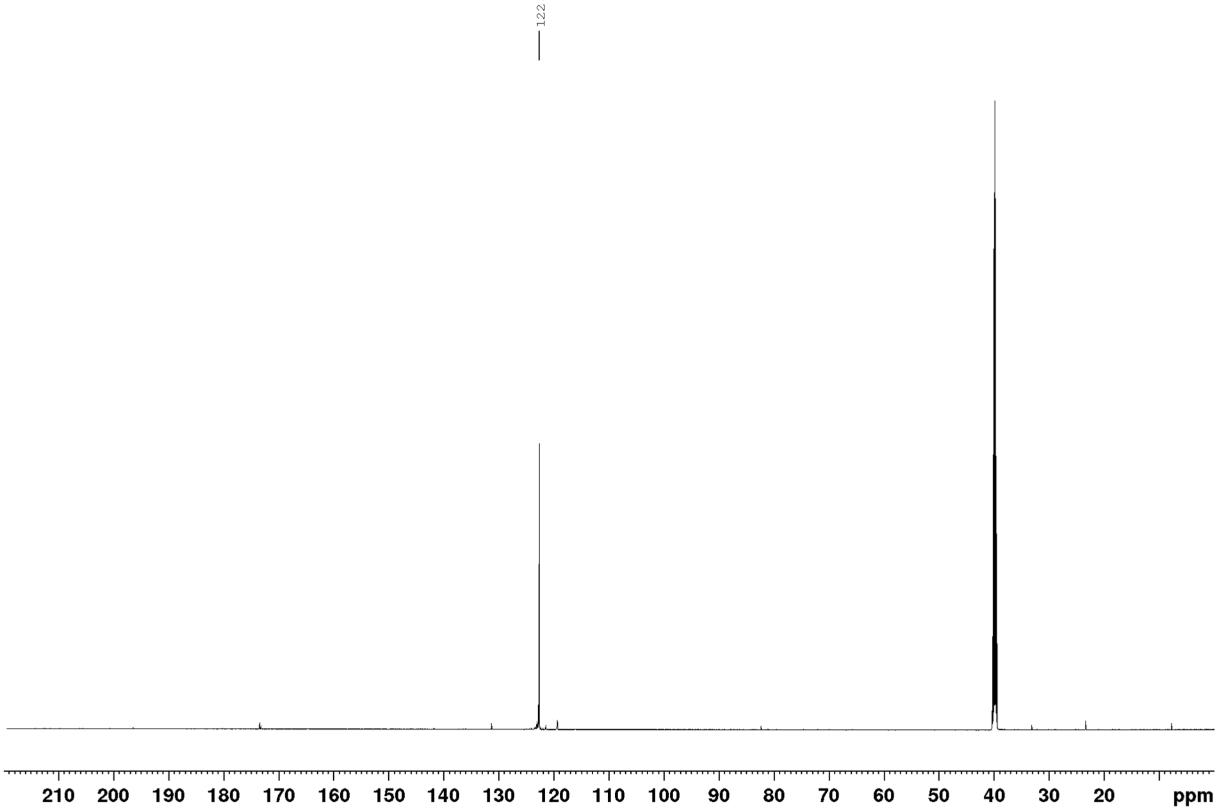

3,4,6-Trideuterio[5-^{13}C]2-(2-oxobutanamido)benzoic acid **17**

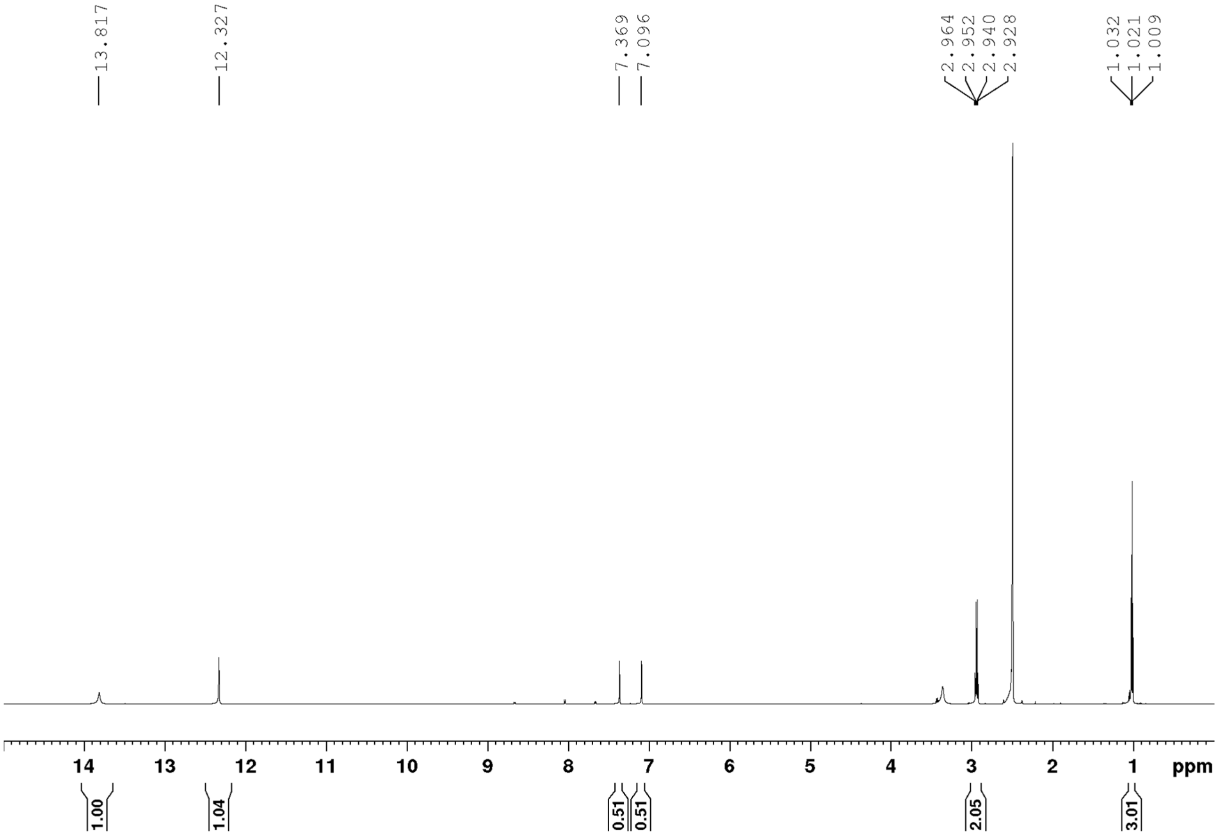

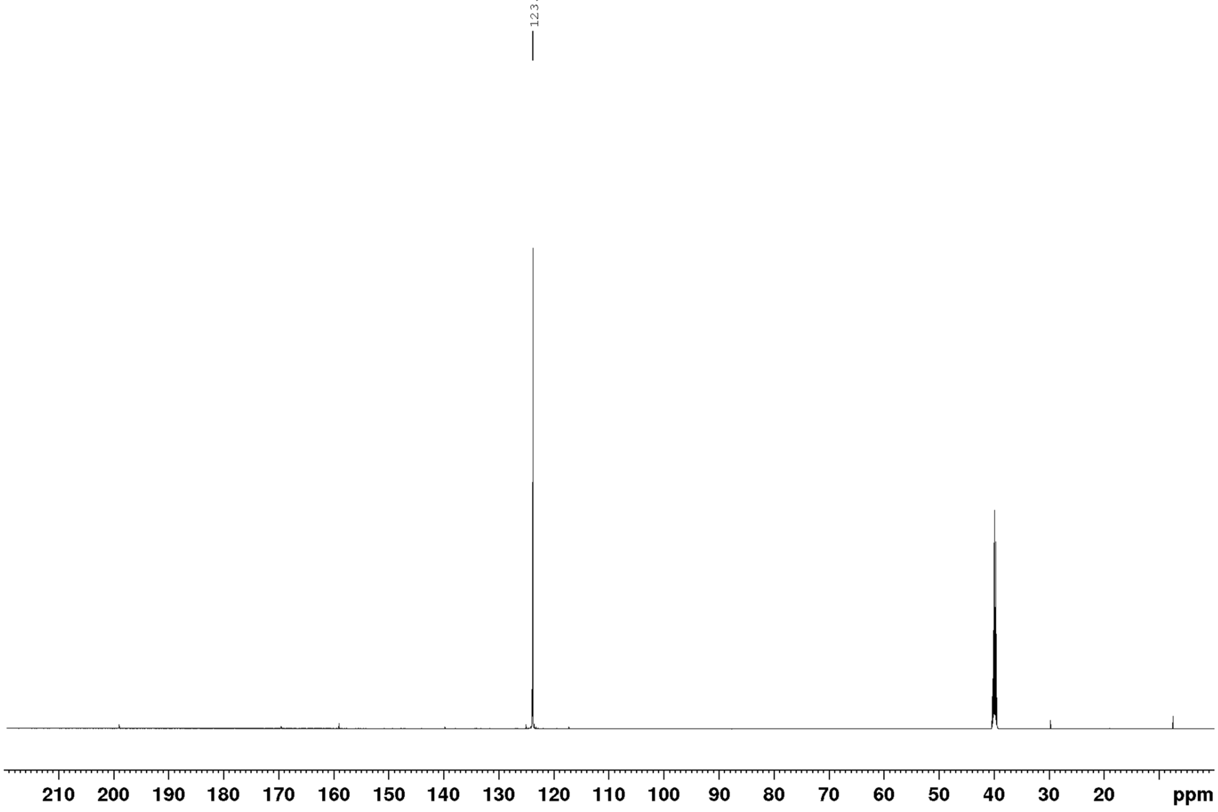

4,6-Dideuterio[5-^{13}C]anthranilic acid **18**

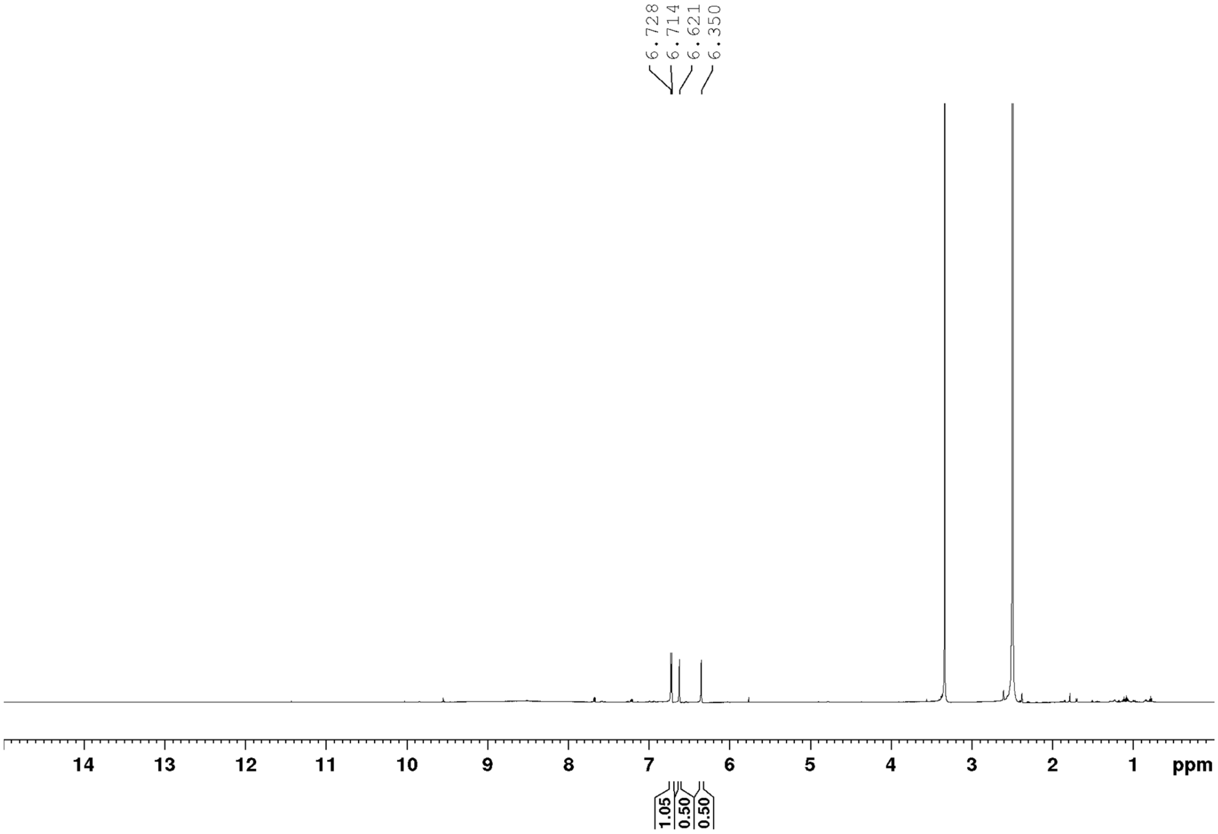

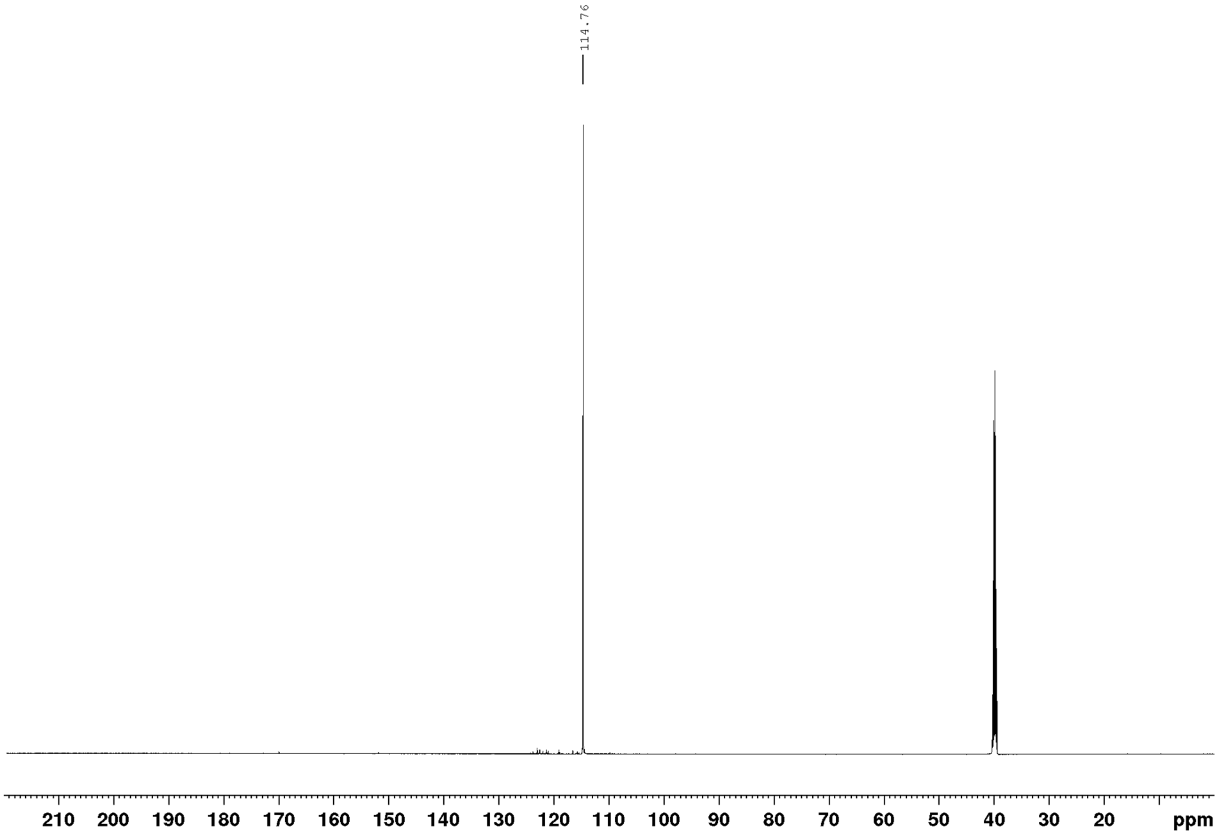

3. Protein overexpression

His-tagged GB1: The pET-M11 plasmid containing the sequence for a his-tagged GB1 protein (H6-GB1) was transformed into an *E. coli* BL21(DE3) strain. Bacterial cultures were grown in LB medium (Luria Broth) at 37 °C until an OD_{600} of 0.7-0.9, then centrifuged at 2000 g for 10 minutes at 20 °C. The pellet was washed with M9 salts buffer (6 g/L Na_2HPO_4, 3 g/L KH_2PO_4, 0.5 g/L NaCl) and again centrifuged at 2000 g for 10 minutes at 20 °C. The pellet was suspended in a small volume (V_{LB} : V_{M9} = 4 : 1) of M9 minimal medium supplemented either with unlabeled NH_4Cl (1 g/L) and increasing amounts of compound **4** (predissolved in H_2O at a concentration of 100 mg/mL) to acquire selectively labeled protein or with $^{15}NH_4Cl$ (1 g/L) to acquire uniformly labeled protein (Marley et al. 2001). The cultures were grown for 45 minutes at 37 °C and induced with 0.8 mM isopropyl-β-D-thiogalactopyranosid (IPTG). Expression was carried out at 30 °C overnight, the cells were harvested by centrifugation at 4000 g for 10 minutes at 4 °C and the pellets were resuspended in 30 mL of buffer A (20 mM sodium phosphate, pH 7.4, 500 mM NaCl, 20 mM imidazole). The cells were lysed by sonication and the lysates were centrifuged at 40000 g for 20 minutes at 4 °C. The supernatant was applied to a Ni^{2+}-charged HisTrap Chelating 5 ml column (GE Healthcare) equilibrated with 5 column volumes (CV) of buffer A. Unbound proteins were washed from the column by 5 CV of buffer A, 3 CV of buffer B (20 mM sodium phosphate, pH 7.4, 1.5 M NaCl, 20 mM imidazole), followed by another 5 CV of buffer A. H6-GB1 was eluted with 5 CV of buffer C (20 mM sodium phosphate, pH 7.4, 500 mM NaCl, 250 mM imidazole) and concentrated to ≈ 1 mM while the buffer was exchanged to buffer D (10 mM sodium phosphate, pH 7.4, 100 mM NaCl). NMR samples contained 1 mM protein and 10 % D_2O.

Brd4-BD1: Recombinant human Brd4-BD1 (bromodomain 1 of Bromodomain containing protein 4) was expressed in *E. coli* BL21(DE3) containing an N-terminal TEV-cleavable His6-tag (the expression plasmid was kindly provided by Boehringer Ingelheim). Uniformly ^{15}N / ^{13}C-tryptophan labeled H6-TEV-Brd4-BD1 was expressed following the expression protocol for efficient isotopic labeling of recombinant proteins using a fourfold cell concentration in M9 minimal medium supplemented with $^{15}NH_4Cl$ (1 g/L) and 3,5-dideuterio[4,6-$^{13}C_2$]anthranilic acid **11** (50 mg/L, predissolved in H_2O at a concentration of 100 mg/mL) (Marley et al. 2001). After resuspension in minimal medium, cells were put for 5 min on ice and 30 min at 18 °C before induction with 0.4 mM IPTG (isopropyl-β-D-1-thiogalactopyranoside). Cells were harvested by centrifugation at 4000 g after 18 hours of expression at 18 °C. The pellet was resuspended in 40ml of buffer containing 20mM sodium phosphate, 500mM NaCl, 20mM imidazole (pH 7.5), 5% glycerol and protease inhibitor (Roche cOmplete Mini, EDTA free). Bacteria were lysed by sonication and the lysates were centrifuged at 40000 g for 20 minutes at 4 °C. Proteins were purified by Ni^{2+} affinity chromatography (HiTrap Chelating HP, 5 mL, GE Healthcare), incorporating a high salt wash (buffer containing 1.5 M NaCl), and cleaved with TEV protease overnight at 4 °C (20 mM sodium phosphate, 500 mM NaCl, 2.5% glycerol, 2 mM β-mercaptoethanol). The cleaved protein was again loaded onto a Ni^{2+} column to bind the cleaved His6-tag and the His6-tagged TEV protease. The flow-through containing Brd4-BD1 was concentrated and loaded onto a gel filtration column (HiLoad 16/60 Superdex 75pg, GE Healthcare). Brd4-BD1 containing fractions were concentrated in an Amicon Ultra-15 centrifugal filter device 3K NMWL and stored at -20°C. NMR samples were prepared in 10 mM sodium phosphate, 100 mM NaCl, pH 7.5 and contained 0.4-3 mM protein and 10 % D_2O. Uniformly $^{15}N/^{13}C$-labeled Brd4-BD1 was overexpressed as described above, but using 1g/L $^{15}NH_4Cl$ and 3g/L $^{13}C_6$-glucose in the corresponding M9 minimal medium.

4. Quantification of H6-GB1 labeling using [^{15}N]anthranilic acid 4

^{1}H 1D and ^{1}H-^{15}N HSQC spectra were acquired at 298 K at 600 MHz for either uniformly ^{15}N-labeled H6-GB1 or selectively labeled H6-GB1. Uniformly ^{15}N-labeled H6-GB1 was expressed using $^{15}NH_4Cl$ as sole nitrogen source, whereas selectively labeled H6-GB1 was expressed using increasing amounts of [^{15}N]anthranilic acid **4** (2, 5, 10, 25, 50, 100, 200 mg/L) and 1 g/L unlabeled NH_4Cl. The HSQC spectra of the selective labeled protein showed only a single peak at δ_H = 10.53 ppm and δ_N = 130.96 ppm (Fig. S1, red spectrum). This peak corresponds to the ^{1}Hε-^{15}Nε side chain spin system of the single tryptophan in H6-GB1 arising from the incorporation of the [^{15}N]anthranilic acid **4** precursor. The peak height corresponding to the signal intensity was normalized to the height of a reference peak in the ^{1}H 1D spectrum at δ_H = -0.42 ppm (Fig. S1b). The normalized signal intensities were expressed as percent of protein saturation, where the normalized signal of the ^{1}Hε-^{15}Nε side chain spin system of uniformly ^{15}N-labeled H6-GB1 was chosen as 100 % saturated. The resulting values (%) were displayed against the concentration of [^{15}N]anthranilic acid **4** in the *E. coli* overexpression medium (main article; Fig. 1b). An exponential saturation function was plotted through the data points in the form of y = A1 * exp(x/t1) + y0.

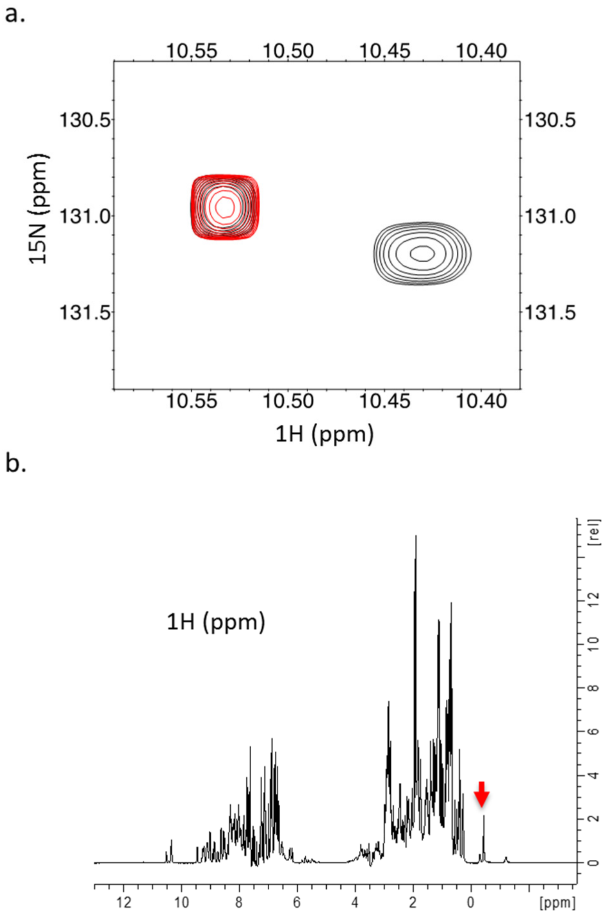

Fig. S1: a. Section of ^{1}H-^{15}N HSQC spectra of uniformly ^{15}N-labeled (black spectrum) and selectively labeled *His-tagged GB1* (red spectrum). The red peak corresponds to the side chain amine of tryptophan arising from the incorporation of [^{15}N]anthranilic acid **4** at 50 mg/L precursor concentration in the expression medium; b. ^{1}H 1D reference spectrum of *His-tagged GB1*. The peak height of the peak at -0.42 ppm (red arrow) was used to reference the signal intensity obtained from the HSQC spectrum.

5. NMR spectra of selectively Trp-^{13}C-labeled and ^{13}C-uniformly labeled Brd4-BD1

All spectra were acquired at 298 K at 600 MHz on 300 µM samples containing 10 mM sodium phosphate, pH 7.5, 100 mM NaCl and 10 % (v/v) D_2O. For each sample, i) Trp-selectively ^{15}N-fully labeled Brd4-BD1 (using 3,5-dideuterio [4,6-$^{13}C_2$]anthranilic acid **11** and 1g/L $^{15}NH_4Cl$ during the expression), ii) uniformly ^{15}N/^{13}C-labeled Brd4-BD1 (using 1 g/L $^{15}NH_4Cl$ and 3 g/L $^{13}C_6$-glucose) and iii) uniformly ^{15}N-labeled Brd4-BD1 (using 1 g/L $^{15}NH_4Cl$), two non-constant time ^{1}H-^{13}C HSQC spectra for the aromatic and aliphatic region, a ^{1}H-^{15}N plane of an HNCO, a ^{1}H-^{15}N HSQC and a ^{1}H 1D spectrum were recorded.

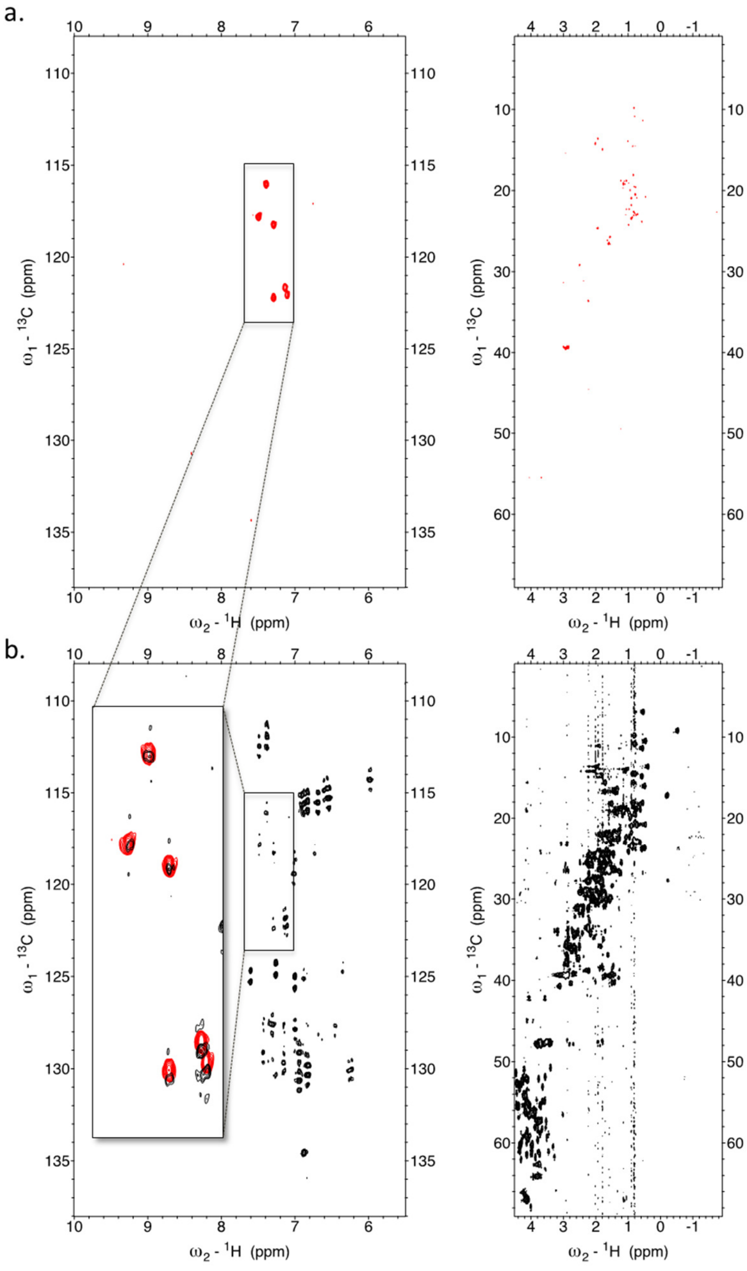

Fig. S2: a. Aromatic and aliphatic regions of uniformly ^{15}N / selectively Trp-^{13}C-labeled Brd4-BD1; b. Aromatic and aliphatic regions of uniformly ^{15}N/^{13}C-labeled Brd4-BD1 (black); an overlay with the spectrum of the selectively ^{13}C-Trp labeled sample is shown in red.

The HNCO planes for the Trp-^{13}C-selectively labeled or ^{15}N-labeled samples were completely devoid of resonances, confirming that the precursor does not label carbonyl-carbons of the protein and therefore suggesting no isotope scrambling (data not shown).

Figure S2 shows the aromatic and aliphatic regions of Trp-selectively labeled Brd4-BD1 using compound **11** (Fig. S2a) and uniformly ^{15}N/^{13}C-labeled Brd4-BD1 (Fig. S2b). As anticipated and described in the main text, the ^{1}H-^{13}C HSQC spectra of the Trp-selectively labeled Brd4-BD1 reveal only the 6 signals, caused by the ^{13}C-^{1}Hε3 and ^{13}C-^{1}Hη2 spin pairs of the three tryptophan residues present in the protein. An overlay of this spectral region with the equivalent region of fully labeled Brd4-BD1 shows that the corresponding ^{13}C resonances in the fully labeled background are severely affected by the strong scalar coupling to neighboring ^{13}C nuclei. Furthermore, the signal intensities are relatively weak due to splittings and line shape distortions. Interestingly, as anticipated from the isotope pattern of precursor **11**, the deuterium isotope shift in the spectrum of the selectively Trp-labeled sample (fig. S2b red) is larger for the ^{13}C-^{1}H$_{\eta 2}$ position than for the ^{13}C-^{1}H$_{\varepsilon 3}$ position.

Similar spectra were also recorded for uniformly ^{15}N-labeled Brd4-BD1 in order to estimate the contribution of ^{13}C natural abundance to the spectra. The spectrum for the aliphatic region shows the same weak signals as the spectrum of Trp-^{13}C-selectively labeled Brd4-BD1, confirming that the precursor is solely incorporated into tryptophan residues (Fig. S3b & S3c). Figure S3a shows the corresponding spectrum of the ^{15}N/^{13}C-fully-labeled Brd4-BD1 at the same contour level for comparison.

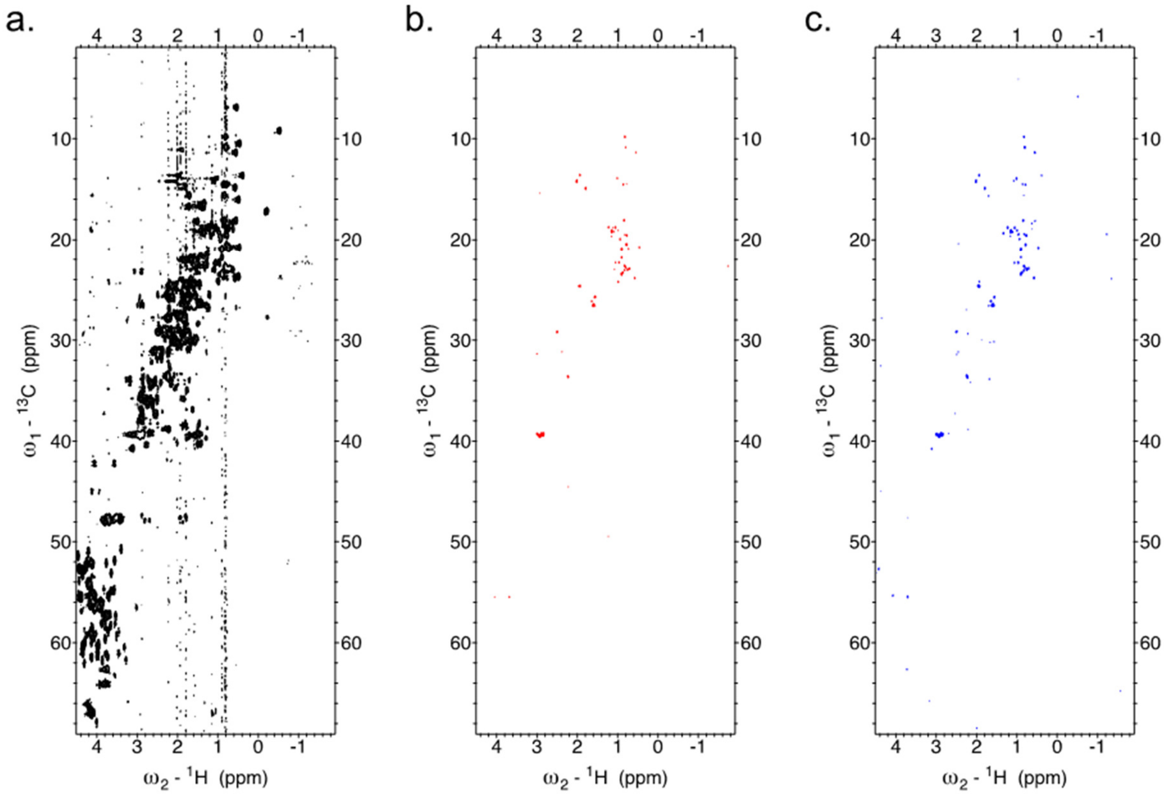

Fig. S3: Aliphatic regions of ^{1}H-^{13}C HSQC spectra of a. uniformly ^{15}N/^{13}C-labeled Brd4-BD1; b. uniformly ^{15}N / selectively Trp-^{13}C-labeled Brd4-BD1; c. uniformly ^{15}N-labeled Brd4-BD1.

Furthermore, amide spectra prove the incorporation of anthranilic acid into tryptophan residues. Figure S4 shows an overlay of ^{1}H-^{15}N HSQC spectra of uniformly ^{15}N / selectively Trp-^{13}C-labeled Brd4-BD1 and uniformly ^{15}N-labeled Brd4-BD1. The three resonances stemming from the Trp-$N_{\varepsilon 2}$ group of the three Trp residues are missing in the spectrum of the selectively Trp-^{13}C-labeled Brd4-BD1, given that the precursor 3,5-dideuterio [4,6-$^{13}C_2$]anthranilic acid **11** carries an unlabeled amino-group.

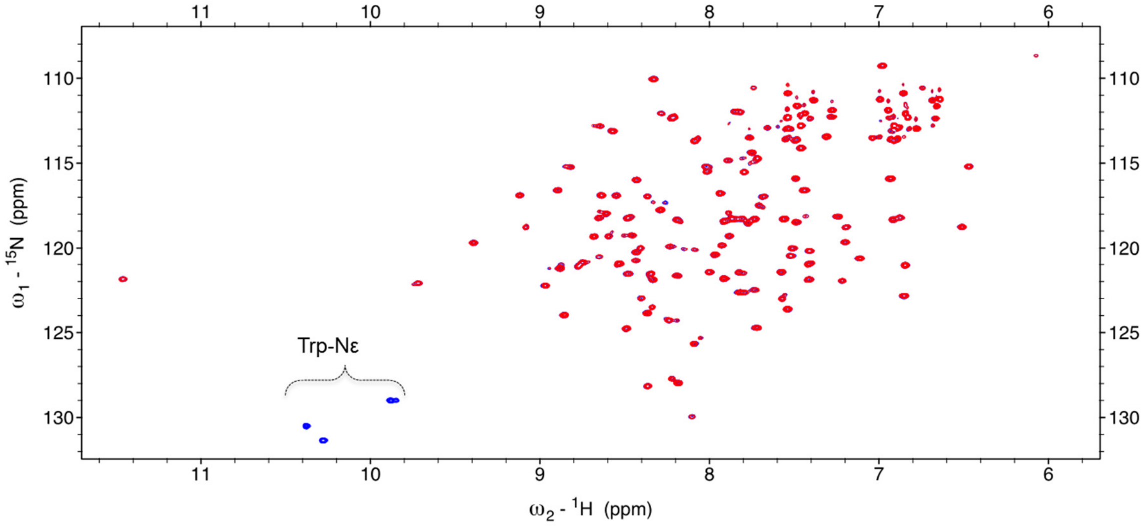

Fig. S4: Overlay of ^{1}H-^{15}N HSQC spectra of uniformly ^{15}N / selectively Trp-^{13}C-labeled Brd4-BD1 (red) and uniformly ^{15}N-labeled Brd4-BD1 (blue).

6. Literature

1 Hijji YM, Ellis B (2003) Microwave Assisted Efficient Conversion of Anhydrides to Cyclic Imides and N- Methoxy Imides. ECSOC-7 E010.

2 Liu Z, Quan Q, Wang W (2009) Biosynthesis of [1-^{15}N] L-tryptophan from ^{15}N labeled anthranilic acid by fermentation of *Candida utilis* mutant. Amino Acids, 36: 71-73.

3 Kafka S, Proisl K, Kašpárková V, Urankar D, Kimmel R, Košmrlj J (2013) Oxidative ring opening of 3-hydroxyquinoline-2,3(1*H*,3*H*)-diones into *N*-(α-ketoacyl)anthranilic acids. Tetrahedron, *69*: 10826-10835.

4 Lichtenecker RJ (2014) Synthesis of aromatic $^{13}C/^{2}H$-α-ketoacid precursors to be used in selective phenylalanine and tyrosine protein labelling. Org Biomol Chem 12:7551-7560.
